# Cancer-associated fibroblasts at the crossroads of tumor progression and therapy resistance: from heterogeneity to precision reprogramming

**DOI:** 10.1186/s43046-025-00334-7

**Published:** 2025-12-22

**Authors:** Ravi Adusumalli, Rajkiran Reddy Banala

**Affiliations:** 1 Department of Biosciences, University of Oslo, Oslo, 0316 Norway; 2 Arthur JE Child comprehensive Cancer center, Alberta Health Services, Calgary, AB T2N 5G2, Alberta, Canada

**Keywords:** Cancer-associated fibroblasts (CAFs), Tumor microenvironment (TME), Fibroblast heterogeneity, Immune evasion, Therapy resistance, Stromal reprogramming, Precision oncology

## Abstract

Cancer-associated fibroblasts (CAFs) are pivotal regulators of the tumor microenvironment (TME), driving malignancy through extracellular matrix remodeling, paracrine and metabolic crosstalk, angiogenesis, fibrosis, and immune suppression. Emerging single-cell and spatial multi-omics have revealed CAF heterogeneity and plasticity, with subtypes such as myofibroblastic, inflammatory, antigen-presenting, and metabolic CAFs exerting context-dependent functions that can either promote or restrain tumor growth. This duality cautions against indiscriminate stromal ablation and highlights the need for precision strategies. CAFs also mediate resistance to chemotherapy, radiotherapy, targeted agents, and immunotherapy by creating physical and biochemical barriers and fostering immune exclusion. Therapeutic approaches span depletion strategies, pathway inhibitors, and stromal reprogramming using vitamin D receptor agonists, retinoids, and epigenetic modulators, often in combination with immunotherapies. However, CAF plasticity and the lack of exclusive markers remain major challenges. This review positions CAFs as dynamic regulators of cancer hallmarks and argues for a paradigm shift toward precision stromal oncology, where the trajectory from CAF depletion to CAF reprogramming and CAF-guided combinatorial therapies reshapes cancer treatment itself.

**Supplementary Information:**

The online version contains supplementary material available at 10.1186/s43046-025-00334-7.

## Introduction

Cancer is being more widely understood not just as a disease of malignant cells, but as a complex multicellular ecosystem where stromal and immune systems play a crucial role in regulating all stages of tumorigenesis [1, 2]. Cancer cells and the tumor microenvironment (TME) interact in a multifaceted and bidirectional manner, influencing tumor growth, progression, metastasis, and the response to treatment. Comprehending these dynamic interactions is essential for the advancement of cancer research and therapy. The TME constitutes a dynamic ecosystem characterized by the reciprocal interactions between non-malignant stromal cells, such as immune cells, endothelial cells, and fibroblasts, and cancer cells. These interactions are pivotal in driving the initiation, progression, and resistance to therapy of the disease. Furthermore, stromal elements create mutual interactions with cancerous cells that significantly influence tumor behaviour [1, 3–5]. Thus, the progression of cancer is determined not merely by genetic and epigenetic variations within tumor cells, but also by the multifaceted and context-dependent TME.

Among the components of the stroma, cancer-associated fibroblasts (CAFs) stand out as one of the most prevalent and adaptable groups. CAFs are characterized as fibroblastic cells found within tumors that take on an activated phenotype, which is different from their inactive counterparts in healthy tissues. This activation grants myofibroblastic properties, sustained inflammatory signaling, and transformed secretomes. Collectively, these modifications enable CAFs to shape the biochemical, mechanical, and immunological landscape of the tumor [5–7]. Rising from resident tissue fibroblasts, mesenchymal stem cells, epithelial or endothelial cells that are undergoing mesenchymal transition, and even hematopoietic progenitors. This illustrates their remarkable plasticity and functions that are specific to tumors [8, 9]. CAFs are identified by the expression of specific markers, which include α-smooth muscle actin (α-SMA), fibroblast activation protein (FAP), and platelet-derived growth factor receptors (PDGFRs) [10, 11].

Functionally, CAFs reshape the extracellular matrix (ECM), producing growth factors and cytokines, and managing angiogenesis, thereby fostering a pro-tumorigenic niche [12, 13]. In addition, CAFs are notably diverse and adaptable, with a range of origins, transcriptional states, and functions that are contingent upon the TME [14, 15]. This heterogeneity is the foundation of their complex functions in cancer progression, including the mediation of therapy resistance through physical barriers, paracrine signaling, and metabolic reprogramming [16, 17]. In a paradoxical manner, specific CAF populations may also limit the growth of tumors, emphasizing the multifaceted role of CAFs in the progression of cancer [18, 19]. Notable findings include the identification of MHC-II⁺ antigen-presenting CAFs [20], the discovery of conserved spatial CAF neighborhoods across multiple cancers [15], and the evidence of dynamic plasticity that allows CAFs to transition between states in response to cues derived from tumors or therapeutic measures [21–24]. In summary, these discoveries highlight heterogeneity and plasticity as essential principles of CAF biology.

Considering these complex roles, CAFs have drawn significant interest as both biological agents of malignancy and possible therapeutic targets. However, their paradoxical nature, which can either promote tumor progression or inhibit it in different contexts, creates substantial challenges for therapeutic use [18, 19]. Recent progress in single-cell and spatial multi-omics has disclosed distinct subsets of CAFs, each contributing uniquely to tumor progression, immune evasion, and the response to therapy [14, 15, 20]. These conclusions emphasize the importance of developing therapeutic methods tailored to specific subtypes.

Although these progressions have been made, several fundamental questions still lack answers in CAF biology: Which molecular signals are responsible for determining CAF subtype specification and their plasticity? How do CAF-mediated immune and metabolic networks work together to foster resistance to targeted therapies and immunotherapies? Is it possible to safely reprogram CAFs towards phenotypes that restrain tumors rather than depleting them entirely? Addressing these inquiries is vital for utilizing CAF biology to secure enduring clinical benefits. To provide context for these challenges, we propose a conceptual framework that consolidates recent developments in CAF biology with therapeutic translation. This framework, referred to as precision stromal oncology, reconceptualizes CAFs not as uniform targets for elimination, but rather as dynamic and adaptable populations that can be adjusted based on the context [3, 15]. Instead of relying on blunt strategies of indiscriminate stromal depletion, precision stromal oncology proposes a trajectory: (i) initial attempts at CAF depletion, which revealed paradoxical tumor acceleration and systemic toxicity [18, 19]; (ii) new strategies for CAF reprogramming, which normalize fibroblast function and preserve stromal integrity [25, 26]; and (iii) a future of CAF-guided combinatorial therapies, where subtype-specific targeting and biomarker-guided stratification integrate stromal modulation with immunotherapy or targeted agents [27, 28]. Positioning CAF biology within this trajectory offers a roadmap for the subsequent sections, transitioning from mechanistic insights to translational opportunities.

**Table:** Box 1

The AI-driven CAF taxonomy is a reproducible classification of CAF states and niches, which is based on integrated multi-omic data, including single-cell transcriptomics, spatial transcriptomics, proteomics, and metabolomics, as well as imaging data. It leverages machine learning to (i) define robust subtypes, (ii) model the transitions of states and their plasticity, and (iii) generate compact biomarker signatures that are clinically deployable and predict responses to therapies that modify the stroma [3, 20, 23, 29]. Recent innovations in computational biology have started to confront CAF heterogeneity by leveraging integrative, AI-driven frameworks that synthesize multi-omic and spatial data [3, 29]. The application of unsupervised clustering techniques, like Louvain and Leiden community detection, hierarchical clustering, and non-negative matrix factorization, has shown effectiveness in analyzing single-cell RNA-seq datasets to differentiate transcriptionally distinct CAF subsets across a range of tumor types [29]. Concurrently, the application of deep neural network autoencoders and random forest classifiers has been implemented to integrate transcriptomic, proteomic, and spatial features, thereby enabling the prediction of CAF state transitions and lineage plasticity [3, 30]. These methodologies have resulted in several clinically significant biomarker panels, including an ECM stiffness index associated with immune-exclusion phenotypes in pancreatic cancer, a metabolic-coupling signature that reflects CAF-tumor redox interdependence, and an immune-exclusion module that forecasts the response to checkpoint blockade [29–31]. Together, these AI-augmented taxonomies progress from simple descriptive classifications to clinically relevant stromal fingerprints, potentially aiding in the creation of precision combination therapies aimed at both cancer and its supportive stroma [3, 30].	

To implement precision stromal oncology effectively, the biology of CAFs should be approached as a systems-level challenge, incorporating single-cell, spatial, proteomic, and metabolic data into predictive models that can be acted upon clinically [3, 20]. A CAF taxonomy powered by AI, originating from integrated multi-omic and imaging datasets, will be necessary to clarify subtype definitions, deduce state transitions, and produce streamlined biomarker panels for the purpose of patient stratification [29]. This strategy integrates high-resolution discovery methods, specifically single-cell and spatial omics, with machine-learning models that recognize clinically pertinent signatures like immune exclusion, ECM stiffness, and metabolic coupling, and forecasts responses to CAF-modulating interventions [23, 24]. Framing CAFs in this way advances the field from merely descriptive atlases to mechanistic, testable hypotheses that are suitable for translation into adaptive clinical trials.

### Scope of this review

This review intends to present an extensive analysis of CAF biology within the TME, concentrating on their origins, heterogeneity, and their dual roles in tumor progression and suppression. Specifically, this review will: (i) summarize the origins and essential biology of CAFs, providing the necessary context for mechanistic interpretation; (ii) synthesize recent findings from single-cell and spatial research that define the states and neighbourhoods of CAFs; (iii) detail the primary mechanisms by which CAFs remodel the ECM, regulate immune responses, and influence angiogenesis and metabolism; (iv) evaluate strategies for translation that focus on targeting or reprogramming CAFs, including imaging and theranostic techniques, cell-based and molecular therapies, and stromal reprogramming agents; and (v) identify significant knowledge gaps that need to be addressed to translate stromal biology into precision therapies. By incorporating the latest developments in single-cell and spatial biology, we emphasize novel possibilities for precision therapies and assess outstanding challenges in stromal oncology, with the goal of enhancing the conceptual framework for CAF research and motivating creative therapeutic approaches.

### CAFs in the tumor microenvironment

Beyond their extensive presence in the tumor stroma, CAFs are now acknowledged as influential regulators of tumor development. Their heterogeneity, specialized functions, and plasticity are key factors that enable them to reshape the tumor environment, support immune evasion, and alter therapeutic responses [3, 5].

### Origin and heterogeneity of CAFs

**Fig. 1:**
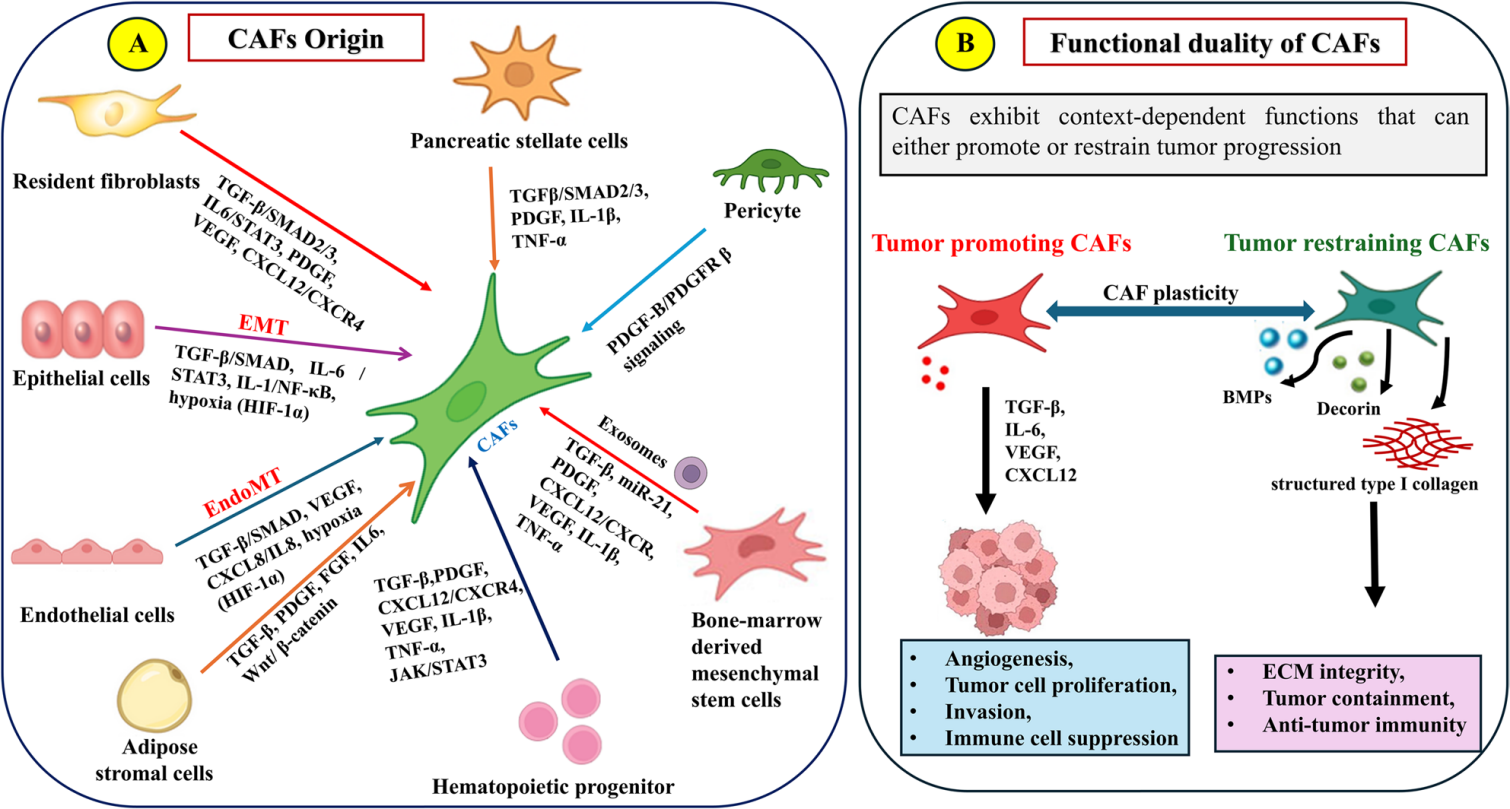
Schematic representation of the diverse cellular sources and functional duality of CAFs. **A** Origins and mechanistic recruitment of CAFs. CAFs consist of a diverse and intricate group of cells, potentially linked to their various origins. A multitude of cells can be activated and recruited as precursors to CAFs, including (1) resident fibroblast cells, (2) pancreatic stellate cells, (3) pericytes, (4) bone-marrow-derived mesenchymal stem cells (BMSCs), (5) hematopoietic stem cells, (6) adipose stromal cells, (7) endothelial cells (via the endothelial-mesenchymal transition; EndMT), and (8) epithelial cells (via the epithelial-mesenchymal transition; EMT). **B** Functional duality and tumor-type-specificity of CAFs. CAFs display context-dependent duality, functioning either as tumor-promoting or tumor-restraining elements depending on their origin and the tumor microenvironment. In pancreatic ductal adenocarcinoma (PDAC), stellate-cell-derived CAFs secrete TGF-β, IL-6, VEGF, and CXCL12, promoting angiogenesis, invasion, and immune suppression. In contrast, breast-cancer CAFs from fibroblast or adipose origins release BMPs, decorin, and structured collagen that preserve ECM integrity and restrain tumor expansion. The functional plasticity of CAFs is highlighted by their capacity to interconvert between tumor-promoting and tumor-restraining phenotypes under distinct signaling and microenvironmental cues [3, 5, 18, 32–40]

CAFs are among the most plentiful and functionally varied stromal cell populations found within the TME. The exploration of their origin and heterogeneity is vital for understanding the dynamics between tumor and stroma, as well as their therapeutic vulnerabilities. Unlike past interpretations that viewed CAFs as a consistent, myofibroblast-like population, recent investigations through single-cell and spatial multi-omics techniques have disclosed a variety of CAF states, each with its own developmental origins, phenotypes, and plasticity [15, 20]. The activation and recruitment of diverse precursor populations, which encompass resident fibroblasts, pancreatic stellate cells, pericytes, bone-marrow-derived mesenchymal stem cells (BMSCs), adipose stromal cells, and endothelial and epithelial cells are facilitated by a coordinated network of feedback signals from tumors and stroma [3, 5]. However, CAFs emerge from multiple cellular origins (Fig. 1), reflecting both local tissue adaptation and systemic contributions to the tumor stroma.

Across the cellular origins illustrated in Fig. 1A, transforming growth factor-β (TGF-β) continues to be the key inducer of fibroblast activation, starting SMAD2/3-dependent transcriptional processes that enhance the expression of α-smooth-muscle actin, fibroblast activation protein, and elements of the extracellular matrix [5, 10, 38, 41]. Platelet-derived growth factor (PDGF) and CXCL12/CXCR4 chemokine signaling additionally enhance fibroblast proliferation and direct migration towards the tumor mass [2, 5, 42], while IL-6/STAT3 and NF-κB pathways reinforce the inflammatory CAF phenotype, which is marked by the secretion of cytokines and chemokines [16, 43, 44]. Simultaneously, the stabilization of hypoxia-inducible factor-1α (HIF-1α) in low-oxygen conditions, along with mechanotransduction through integrin-FAK-YAP/TAZ signaling, sustains the activation of CAFs by linking metabolic stress to cytoskeletal remodeling [45–49]. Furthermore, exosomes originating from tumors that are rich in TGF-β, miR-21, and various regulatory RNAs transform bone-marrow-derived mesenchymal stem cells and pericytes into cells resembling CAFs, thereby connecting systemic inflammatory signals to the remodeling of local stroma [8, 9, 32, 50]. Together, this preliminary signaling events form a persistent feed-forward circuit that upholds CAF heterogeneity and plasticity within the advancing tumor microenvironment [6, 7, 36, 40]. As illustrated in Fig. 1B, the predominance or equilibrium of these signals influences the functional polarity of CAFs: in pancreatic ductal adenocarcinoma (PDAC), prolonged TGF-β and IL-6 signaling in fibroblasts derived from stellate cells fosters a stroma that supports tumors, is fibrotic, and immunosuppressive. In contrast, in breast cancer, fibroblast- and adipose-derived CAFs that are subjected to BMP- and decorin-mediated ECM signals can uphold structural integrity and exhibit tumor-restraining characteristics [18, 19]. This context-sensitive plasticity establishes a mechanistic foundation for the dual functions of CAFs and transitions smoothly into the diversity of their subtypes, which will be discussed in the subsequent sections.

**Fig. 2:**
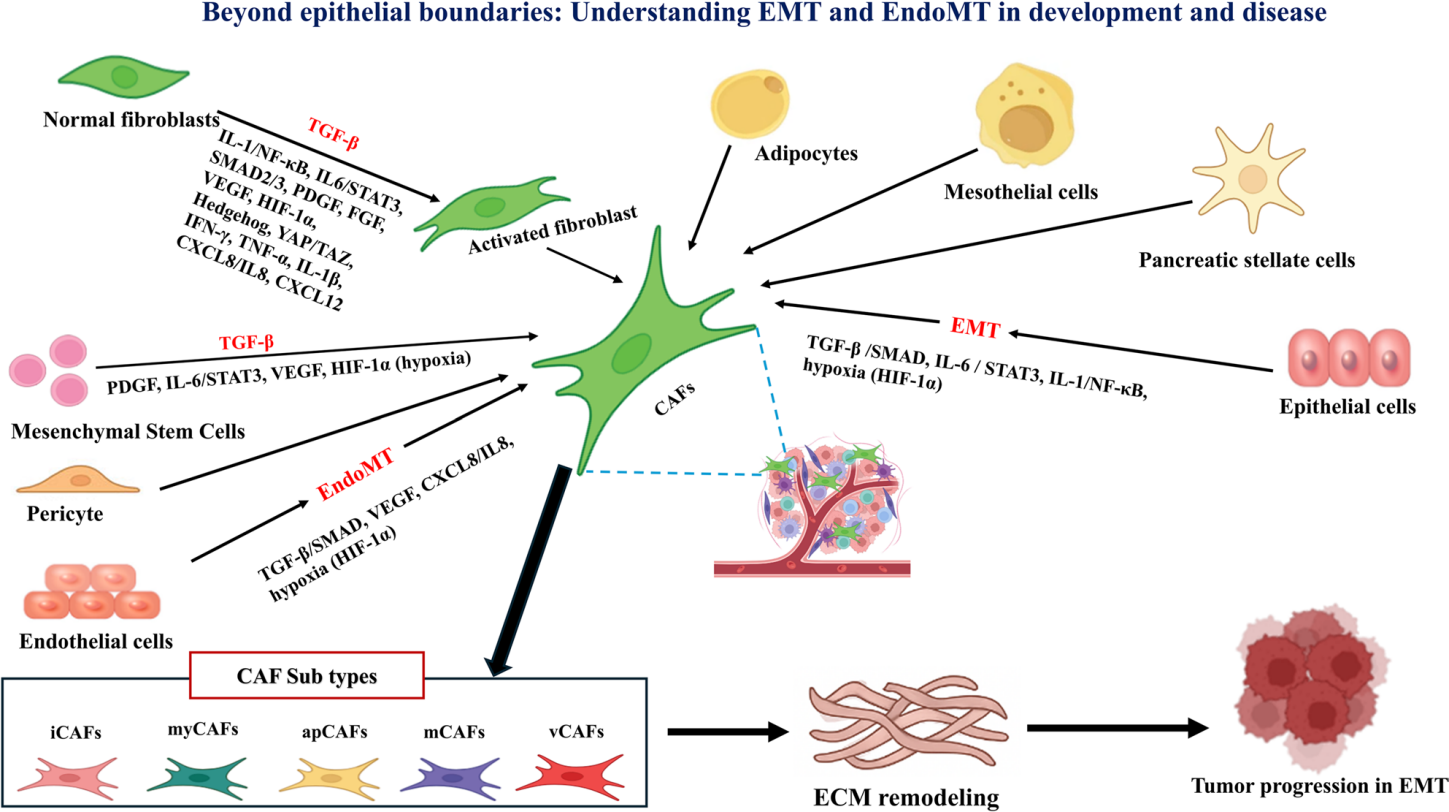
The interplay of tumor-associated macrophages, extracellular matrix proteins, and CAFs in Tumorigenesis. The schematic illustration demonstrates the CAFs comprise a diverse population of cells with distinct subtypes, represented by different colors, highlighting their varied origins and functional heterogeneity. The complex interactions between tumor-associated macrophages (TAMs), extracellular matrix (ECM) proteins, and cancer-associated fibroblasts (CAFs) play a crucial role in oncogenesis. This multifaceted axis contributes to the development and progression of cancer through various mechanisms [9, 16, 38, 39, 41, 43, 51]. Abbreviations: Epithelial-to-mesenchymal transition (EMT), Endothelial-to-mesenchymal transition (EndoMT), Mesenchymal stem cells (MSCs), Transforming growth factor beta (TGF-β), Platelet-derived growth factor (PDGF), Fibroblast growth factor (FGF), Vascular endothelial growth factor (VEGF), Chemokine (C-X-C motif) ligand 8/Interleukin-8 (CXCL8/IL-8), Interleukin (IL), Signal transducer and activator of transcription 3 (STAT3), Hypoxia-inducible factor (HIF), Interferon (IFN), Nuclear factor kappa-light-chain-enhancer of activated B cells

Research on lineage tracing and molecular profiling suggests contributions from: Resident tissue fibroblasts represent a primary source of CAFs, which become activated due to tumor-derived signals and chronic inflammation [5, 14]. However, CAFs can originate from mesenchymal stromal cells that are derived from bone marrow and subsequently recruited into the TME [9]. This includes the processes of epithelial-to-mesenchymal transition (EMT) and endothelial-to-mesenchymal transition (EndoMT), which lead to the emergence of CAF-like populations under conditions of chronic inflammation and fibrosis [41, 51]. In addition, adipose-derived stromal cells are increasingly identified as a reservoir for CAFs in cancers associated with obesity [32]. Pericytes, which typically maintain vascular integrity, may undergo a phenotypic shift towards fibroblast-like cells within tumors. Pericyte-derived CAFs demonstrate increased pro-angiogenic and matrix-remodeling capabilities, thereby connecting vascular biology with stromal remodeling and tumor advancement (Fig. 2) [50, 52].

This multiplicity reinforces the understanding that CAFs are not confined to a single lineage; instead, they represent a functional state shaped by the selective pressures present in the TME.

CAFs exhibit significant variability in gene expression, functionality, and spatial distribution both within and among tumors. Recent studies utilizing single-cell transcriptomics and spatial mapping reveal that CAFs consist of transcriptionally and spatially unique populations that fulfill specialized functions in tumor advancement, immune regulation, and metabolic reprogramming [3, 14, 15]. Crucially, these identities are not static; CAFs exhibit significant plasticity, allowing for dynamic shifts between states in reaction to tumor signals and treatments [33, 53]. The multiple sources and inherent heterogeneity of CAFs lay the foundation for understanding their division into functional subtypes.

Collectively, the various developmental origins and phenotypic characteristics of CAFs emphasize that these cells ought not to be viewed as a homogeneous stromal population, but instead as ecosystem engineers within the TME. Their heterogeneity reflects both developmental processes and the capacity to adapt dynamically to changing tumor environments, immune challenges, and treatment strategies. Recent progress in single-cell and spatial multi-omics has further emphasized this adaptability, indicating that CAF subtypes are commonly arranged spatially and are contingent upon the context [15, 20, 23]. This framework redirects attention from rigid classification to comprehending CAFs as adaptable and multifunctional regulators, which can alter the tumor ecosystem in manners that may either promote or inhibit malignancy.

Beyond these general principles, it is increasingly recognized that the origins of CAFs vary in a cancer-type-specific manner, influenced by the tissue context and oncogenic signaling networks. Emerging research highlights the unique lineage trajectories present in lung, breast, ovarian, colorectal, and pancreatic cancers, where local stromal reservoirs and cues from tumors imprint the identity of CAFs.

### Cancer-type specific origins of CAFs

Furthermore, it is becoming increasingly evident that the origins of CAFs are not fixed but are instead shaped by the specific tumor characteristics and the surrounding microenvironmental signals.

In lung cancer, the predominant source of CAFs is reprogrammed resident fibroblasts that have been influenced by TGF-β and IL-6, with endothelial-to-mesenchymal transition also contributing to their formation. Recent studies utilizing single-cell spatial multi-omics have revealed different CAF subtypes that occupy distinct spatial niches and have immunoregulatory programs, which support immune evasion and resistance to therapeutic interventions [15, 54].

In breast cancer, CAFs predominantly originate from adipose-derived stromal cells and tissue fibroblasts that experience chronic activation. Distinct populations of CAFs, including the CD10⁺GPR77⁺ subsets, are known to promote stemness and chemoresistance, while other subsets play a role in modulating endocrine responsiveness in luminal tumors [32, 34, 35]. These findings underscore how local stromal reservoirs dictate CAF diversity.

In ovarian cancer, peritoneal mesenchymal cells and omental fibroblasts serve as the main precursors. Studies have revealed that signaling through platelet-derived growth factor (PDGF) is essential for maintaining CAF activation and strengthening hypoxia-driven HIF-1α feedback, thus hastening the progression of clear cell ovarian cancer [42]. This highlights the role of paracrine signaling loops in determining organ-specific CAF origins.

In colorectal cancer (CRC), fibroblasts influenced by inflammatory and hypoxic stimuli differentiate into tumor-supporting CAFs. Recent findings have revealed that FOS-driven inflammatory CAFs coordinate liver metastasis through the SFRP1–FGFR2–HIF1 axis [55]. Moreover, stromal mediators including IGFBP7 and STC1 act as paracrine drivers of growth and dissemination [56, 57].

In pancreatic ductal adenocarcinoma (PDAC), the main origin of CAFs is attributed to quiescent pancreatic stellate cells (PSCs). Upon their activation, PSCs give rise to various subsets, which consist of myofibroblastic CAFs (myCAFs), inflammatory CAFs (iCAFs), and antigen-presenting CAFs (apCAFs) [20, 36]. This heterogeneity illustrates the lineage plasticity of stellate cells and their essential role in the development of PDAC desmoplasia.

Collectively, these cancer-specific insights highlight that the origin of CAFs is not consistent but rather influenced by the tissue environment, stromal reservoirs, and oncogenic signaling pathways is depicted in Table 1.

**Table 1:** Dominant CAF origins across selected tumor types and their biological implications

Tumor type	Dominant CAF origin	Key notes	References
Lung cancer	Resident fibroblasts; Endothelial-to-mesenchymal transition (EndMT)	IL-6/TGF-β signaling drives CAF activation; single-cell analyses reveal immunoregulatory CAF subsets sustaining immune evasion and therapy resistance.	[15, 16, 54]
Breast cancer	Resident fibroblasts; Adipose-derived stromal cells	CD10⁺GPR77⁺ CAFs promote stemness and chemoresistance; CAFs regulate endocrine responsiveness in luminal tumors; adipose tissue contributes to CAF pool.	[32, 34, 35]
Ovarian cancer	Omental fibroblasts; Peritoneal mesenchymal cells	PDGF-driven CAF activation sustains HIF-1α signaling, promoting hypoxia and tumor progression; CAFs remodel ECM to enable angiogenesis and dissemination.	[42, 58]
Colorectal cancer	Fibroblasts activated by inflammatory/hypoxic cues	FOS-driven CAFs promote liver metastasis via SFRP1–FGFR2–HIF1 axis; stromal IGFBP7 and STC1 enhance paracrine tumor growth and spread.	[55–57]
Pancreatic cancer (PDAC)	Pancreatic stellate cells (PSCs)	PSC activation generates myCAFs, iCAFs, and apCAFs; CAF heterogeneity shapes immune suppression and desmoplasia; PSC reprogramming with vitamin D or retinoic acid restores quiescence.	[20, 25, 26, 36]

*CAF* Cancer-associated fibroblast, *EndMT* Endothelial-to-mesenchymal transition, *PDGF* Platelet-derived growth factor; HIF-1α, hypoxia-inducible factor-1 alpha, *ECM* Extracellular matrix, *FOS* Fos proto-oncogene, *SFRP1 *Secreted frizzled-related protein 1, *FGFR2* Fibroblast growth factor receptor 2, *STC1* Stanniocalcin 1, *IGFBP7* Insulin-like growth factor–binding protein 7, *PDAC* Pancreatic ductal adenocarcinoma, *PSC* Pancreatic stellate cell, *myCAF* myofibroblastic CAF, *iCAF* Inflammatory CAF, *apCAF* Antigen-presenting CAF

### CAF subtype landscape, plasticity, and functional duality

**Fig. 3:**
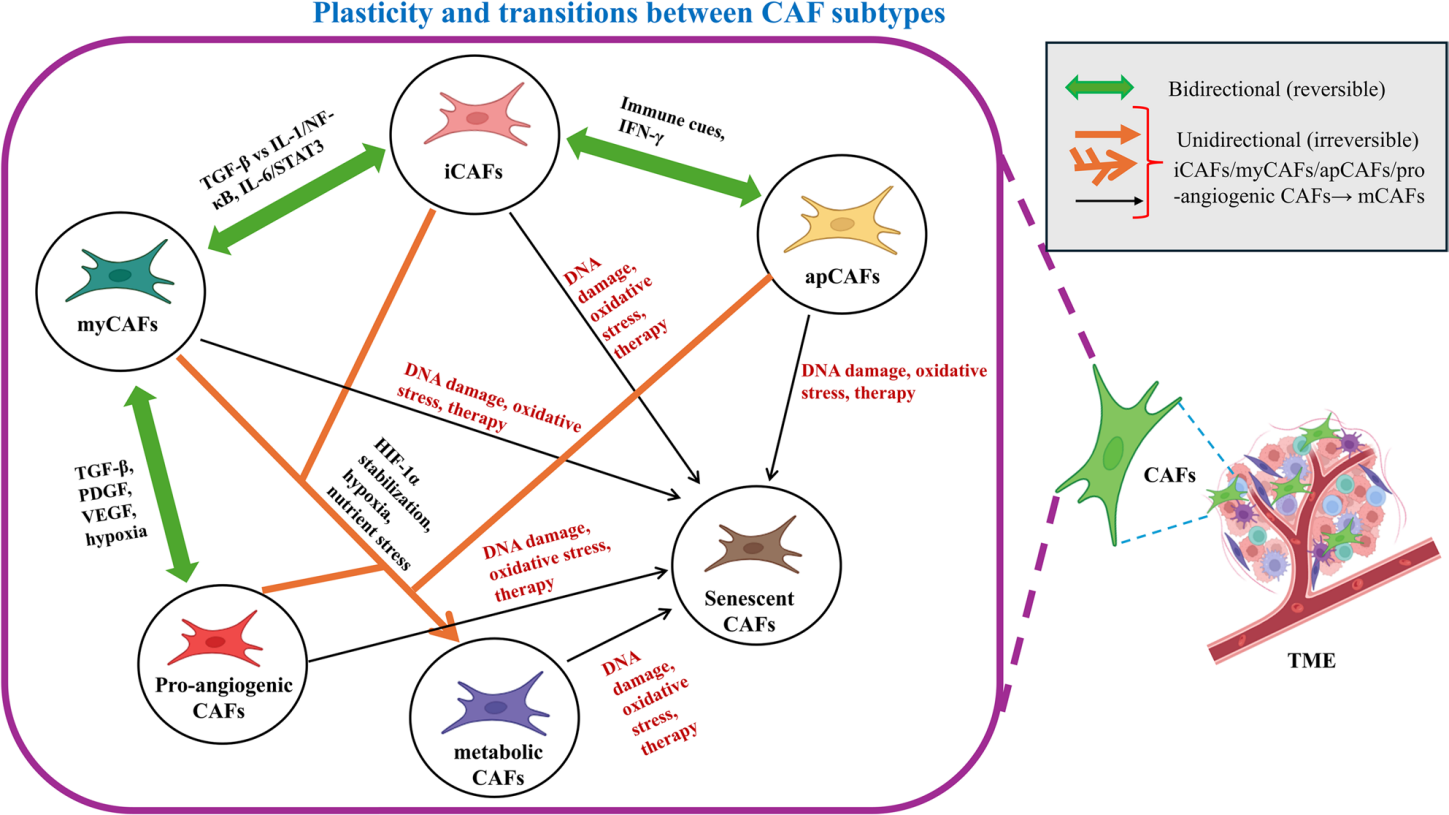
Plasticity and transitions between CAF Subtypes. Heterogeneous CAFs can give rise to various subtypes, including myCAFs, iCAFs, apCAFs, pro-angiogenic CAFs, metabolic CAFs, and senescent CAFs, depending on signals from the tumor microenvironment (TME). These subtypes are not permanent but interconvertible via manipulation of specific signaling pathways. CAF plasticity is complex, and the potential for reversion can vary depending on the specific context and signals present in the TME. Some subsets can revert to a more normal fibroblast state. Schematic representation of bidirectional transitions (↔, green two-headed arrows) driven by specific cues: for instance, iCAFs ↔ myCAFs driven by TGF-β vs. IL-1/NF-κB, IL-6/STAT3; iCAFs ↔ apCAFs driven by immune cues, IFN-γ; myCAFs ↔ pro-angiogenic CAFs driven by TGF-β, PDGF, VEGF, and hypoxia. Unidirectional transitions (→, orange single-headed arrows) include myCAFs/iCAFs/apCAFs/pro-angiogenic CAFs → metabolic CAFs driven by HIF-1α stabilization, hypoxia, and nutrient stress. Additionally, any subtype can transition to senescent CAFs (→, black single-headed arrows) driven by DNA damage, oxidative stress, and therapy respectively [3, 5, 14, 16, 20, 23, 27, 29, 33, 40–43, 53, 55, 59, 60].

CAFs are often organized into various subtypes that are defined by transcriptional programs, molecular drivers, and their spatial distribution. These subtypes represent the functional diversity of CAFs while also emphasizing their potential to interconvert based on the context [3, 14, 15]. This landscape can be simplified into a minimum of four primary states: myCAFs, iCAFs, apCAFs, and metabolically specialized CAFs, each characterized by unique markers and functions as showed in Fig. 3.

### Major CAF subtypes

#### Myofibroblastic CAFs (myCAFs)

Fibroblasts that are contractile and driven by TGF-β/SMAD3, marked by α-SMA, modify the extracellular matrix (ECM), increase the stiffness of tissues, and generate desmoplasia that promotes tumor progression [37, 41].

#### Inflammatory CAFs (iCAFs)

 Cytokine-secreting CAFs activated by IL-1 and NF-κB signaling pathways produce IL-6, CXCL12, and LIF; these factors contribute to the maintenance of chronic inflammation and the creation of an immunosuppressive microenvironment [16, 43].

#### Antigen-presenting CAFs (apCAFs)

A unique MHC-II + population that expresses CD74 while lacking costimulatory molecules; they present antigens in a non-productive way, reducing T cell activation and promoting immune tolerance [3, 20].

#### Metabolic CAFs

Influenced by HIF-1α and metabolic stress to furnish nutrients including lactate, glutamine, and lipids; they aid in metabolic coupling with tumor cells and foster anabolic tumor growth [24, 45].

#### Senescent CAFs

The p16 INK4a + fibroblasts, induced by stress, take on a senescence-associated secretory phenotype (SASP) and secrete cytokines and proteases that facilitate chronic inflammation, angiogenesis, and the advancement of tumors [59].

#### Pro-angiogenic CAFs

CAFs rich in growth factors secrete VEGF, PDGF, and CXCL8; they promote endothelial sprouting, facilitate neovascularization, and play a role in developing resistance to anti-angiogenic therapies [42, 60, 61].

#### Vascular CAFs (vCAFs)

A perivascular subtype that is abundant in programs that promote vessel stabilization and support angiogenesis. This subtype is marked by the presence of NG2/CSPG4, RGS5, and PDGFRβ, and operates at the tumor-vascular interface to influence endothelial interactions, as well as vessel remodeling and stabilization. vCAFs are closely related to angiogenic CAFs, but they form a distinct spatial subset within perivascular niches [60–62].

Even though many CAF subtypes encourage tumor growth, invasion, and immune evasion, increasing evidence points to the possibility that certain CAFs may hinder tumor progression in specific situations as specified in the Table 2.

**Table 2:** CAF subtypes with key drivers, markers, and functional roles

CAF Subtype	Key Drivers	Representative Markers	Functions	References
MyCAFs	TGF-β, SMAD3	α-SMA, TAGLN (Transgelin gene), COL1A1	ECM remodeling, stiffness, desmoplasia	[37, 41]
iCAFs	IL-1, IL-6, NF-κB	IL-6, CXCL12, LIF, Podoplanin (PDPN)	Inflammation, immunosuppression	[16, 43]
apCAFs	IFN-γ, STAT1	MHC-II (HLA-DRA), CD74	Antigen presentation, T cell modulation	[3, 20]
Metabolic CAFs	HIF-1α, lactate signaling	MCT4, GLS1, CPT1A	Nutrient transfer, metabolic coupling	[24, 45]
Senescent CAFs	DNA damage, oxidative stress	p16^INK4a, SA-β-gal, MMPs	Senescence-associated secretory phenotype (SASP), chronic inflammation, fibrosis and progression of cancer	[59]
Pro-angiogenic CAFs	VEGF, PDGF, CXCL8	VEGFA, ANGPTL2, CXCL8	Vascularization, therapy resistance	[42, 60, 61]
Vascular CAFs (vCAF)	PDGF-B/PDGFRβ, angiogenic cues	NG2/CSPG4, RGS5, PDGFRβ	Vessel remodeling, stabilization, angiogenesis	[60–62]

For instance, myCAFs can confine tumor spread by enveloping cancer cells in a dense stroma [18, 19], while some iCAF subsets have been reported to recruit and activate cytotoxic immune cells in defined settings [63]. Likewise, the remodeling of ECM by myCAFs can form physical barriers that hinder vascular invasion, although this same trait also improves resistance to therapy [37]. This duality illustrates that CAF functions are not inherently tumor-promoting; rather, they are determined by subtype identity, spatial localization, and the dynamic interplay with other cells in the tumor microenvironment [59].

A defining characteristic of CAF biology is its plasticity. Subtypes are not static entities; rather, they represent dynamic states that can interchange based on spatial niche, signals derived from tumors, or therapeutic interventions. For instance, iCAFs may convert into myCAFs following extended TGF-β stimulation [14], whereas Hedgehog inhibition or metabolic interventions remodel CAF subpopulations [24, 64]. This adaptability complicates CAF-targeted therapies but also offers an opportunity to reprogram CAFs into less tumor-promoting states [3, 65, 66].

### Spatial context and CAF niches

Building upon the previously described diversity of CAF subtypes, their spatial organization within tumors further determines functional specialization and clinical relevance. Spatial transcriptomics and multi-omics profiling have indicated that CAFs occupy niches that shape tumor evolution and treatment response [15, 23].

Perivascular niches are abundant in vascular CAFs (vCAFs), which play a crucial role in stabilizing newly formed blood vessels and promoting angiogenesis. Their close association with endothelial cells connects them to vascular remodeling and clinical characteristics, including heightened microvessel density and resistance to anti-angiogenic treatments [60, 62]. In contrast, immune niches are predominantly characterized by iCAFs and apCAFs, which either secrete cytokines or present antigens in ways that regulate T cell activity, ultimately affecting the patterns of immune infiltration and the prognosis for patients [67, 68]. Invasive front niches often harbor myCAFs that produce aligned ECM tracks, facilitating the collective movement of tumor cells and correlating with metastatic risk [69].

**Fig. 4:**
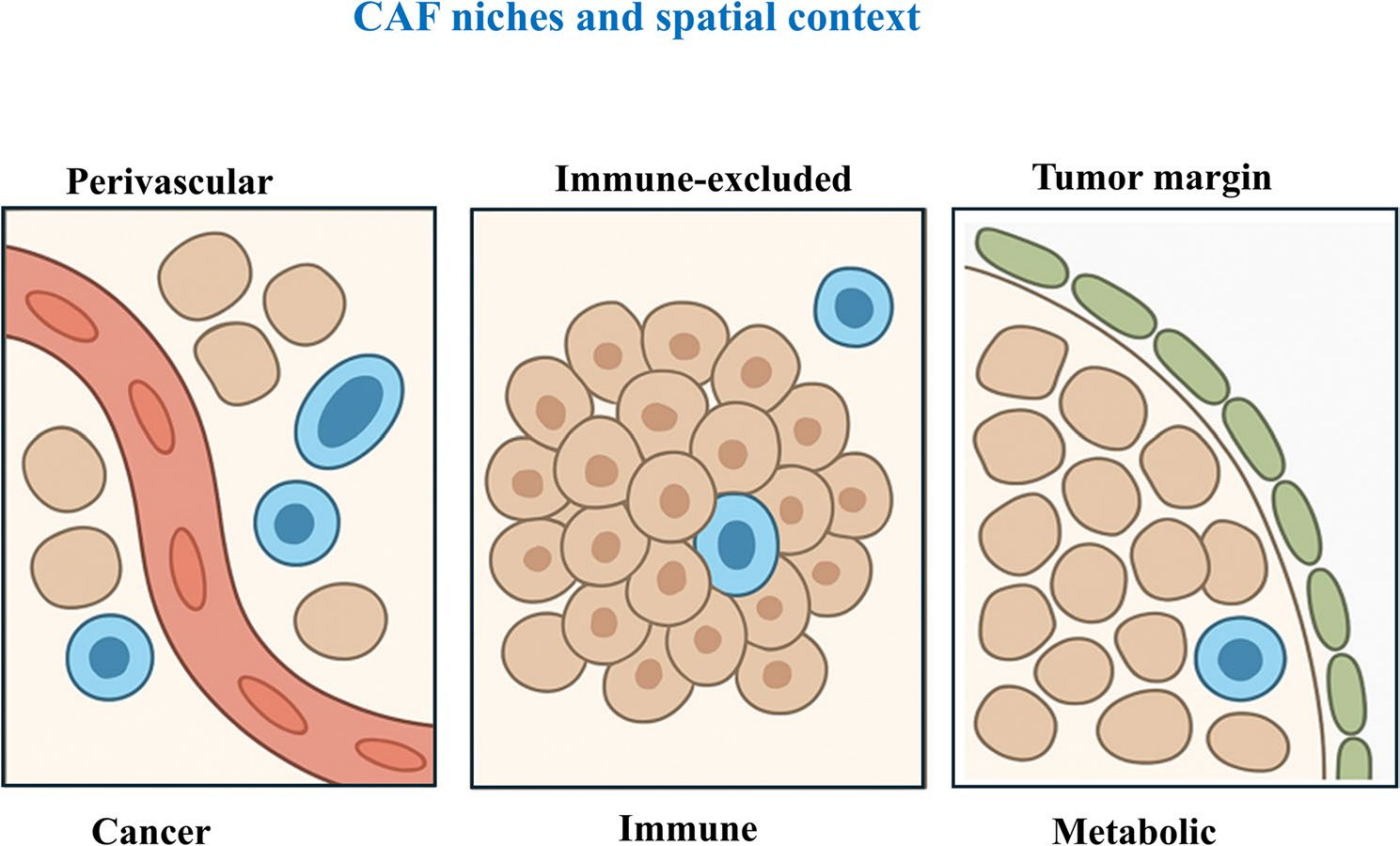
CAF Niches and Spatial Context. Schematic representation of spatially distinct cancer-associated fibroblast (CAF) niches within the tumor microenvironment, illustrating their interactions with cancer cells, endothelial cells, and immune cells. Left (Perivascular niche): CAFs localize adjacent to endothelial cells and blood vessels, where they regulate angiogenesis and vascular permeability. Center (Immune-excluded niche): Dense CAF-rich stroma surrounds clusters of tumor cells, physically excluding cytotoxic T cells and shaping an immunosuppressive barrier. Right (Tumor margin niche): CAFs align at invasive fronts, interacting with cancer cells and immune cells to regulate invasion, immune modulation, and stromal remodeling. Together, these niches highlight the spatial heterogeneity of CAFs and their interactions with cancer cells, endothelial cells, and T lymphocytes [3, 29, 33, 42, 53, 60, 70]

The collective spatial distribution of CAF subtypes forms functionally distinct ecological zones within tumors, as indicated in Fig. 4.

Mapping these niches is of prognostic importance; for instance, the enrichment of perivascular CAFs is linked to poor survival outcomes in ovarian and breast cancers, while clusters of immune-excluding CAFs suggest resistance to checkpoint blockade [58, 71]. Thus, the topography of CAF niches is not only descriptive but also directly associated with clinical outcomes.

#### Fibroblast-to-CAF transition: drivers and regulatory factors

**Fig. 5:**
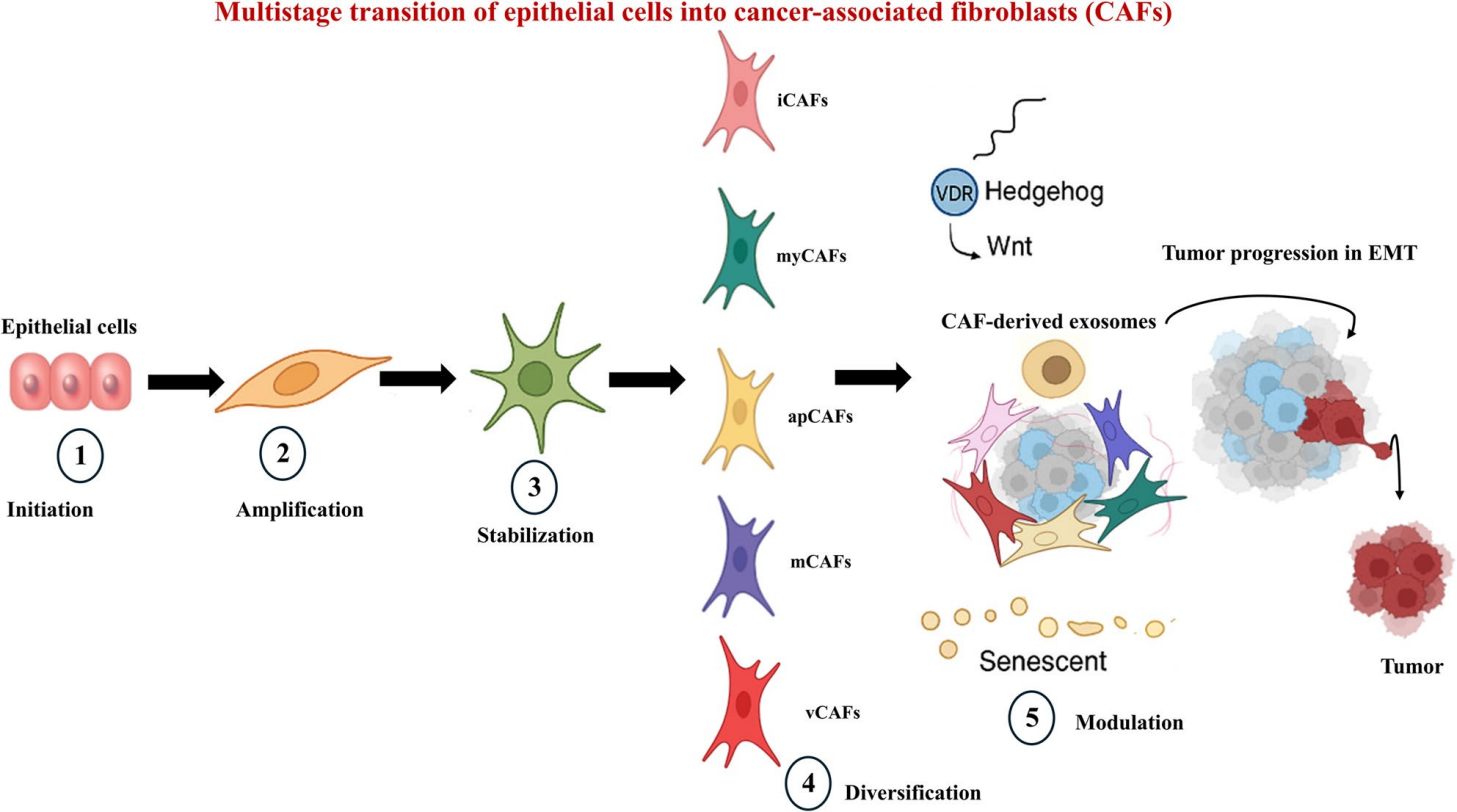
Multistage transition of epithelial cells into CAFs. The diagram illustrates the progressive transformation of epithelial cells into CAFs through five interconnected stages: Initiation (cytokine-driven activation via TGF-β/SMAD, IL-1/NF-κB, IL-6/STAT3) [16, 38, 43], Amplification (paracrine growth factor loops including PDGF and FGF) [42, 60], Stabilization (epigenetic reprogramming and ECM stiffness through integrin–FAK–YAP/TAZ signaling), Diversification (generation of CAF subtypes such as myCAFs, iCAFs, apCAFs, vCAFs, metabolic CAFs, and senescent CAFs under hypoxia and niche-specific cues) [14, 20, 55, 59], and Modulation (reinforcement by Hedgehog, Wnt, and oxidative stress, counter-regulated by VDR and retinoic acid signaling, with CAF-derived exosomes contributing to systemic tumor progression) [36, 40, 72]. Abbreviations: CAF, cancer-associated fibroblast; myCAF, myofibroblastic CAF; iCAF, inflammatory CAF; apCAF, antigen-presenting CAF; vCAF, vascular CAF; ECM, extracellular matrix; TGF-β, transforming growth factor-β; PDGF, platelet-derived growth factor; FGF, fibroblast growth factor; VDR, vitamin D receptor; SASP, senescence-associated secretory phenotype

While spatial organization determines how CAFs operate within the TME; however, their development arises from the dynamic reprogramming of normal fibroblasts. This transition from fibroblasts to CAFs is not a singular event but rather a multistep process that is coordinated by cytokines, growth factors, and stressors present in the TME, as illustrated in Fig. 5.

This development can be understood as comprising five interrelated phases: initiation, amplification, stabilization, diversification, and modulation, with each phase regulated by distinct signaling networks and feedback loops [3, 5].

- **I. Initiation-cytokine signaling: **Early CAF activation is driven by pro-inflammatory and profibrotic cytokines. TGF-β triggers the activation of SMAD2/3, which leads to myofibroblastic differentiation, ECM deposition, and the initiation of immunosuppressive programs [38, 41]. IL-1 activates NF-κB, whereas IL-6 interacts with STAT3, both of which amplify inflammatory signaling and the release of tumor-promoting cytokines [16, 43]. Together, these pathways highlight the first transition from quiescent fibroblasts to activated CAFs with contractile and secretory properties.
- **II. Amplification-growth factors and paracrine loops:** Upon initiation, the activation of CAF is enhanced by growth factors and paracrine interactions with both tumor and immune cells. PDGF significantly encourages the proliferation of CAF and the activation of angiogenic programs, whereas FGF, in specific contexts, aids in proliferative and pro-angiogenic signaling [42]. Sustained IL-6/STAT3 activity fortifies inflammatory CAF conditions, resulting in prolonged immune modulation and enhancing tumor-supportive microenvironments [16, 43].
- **III. Stabilization - epigenetic and extracellular feedback: **Prolonged interaction with signals originating from tumors causes epigenetic modifications, such as histone acetylation and DNA methylation, that reinforce the identity of CAFs and establish a long-lasting “memory” of activation, which continues to exist even after the signals have been withdrawn [39]. At the same time, ECM stiffness and mechanotransduction feedback promote α-SMA expression and contractility, resulting in a self-sustaining cycle of matrix remodeling and CAF persistence [37]. Paracrine feedback from immune cells enhances the stability of CAF phenotypes, integrating them into the tumor stroma [73].
- **IV. Diversification - environmental stressors and niche interactions:** As tumors progress, CAFs differentiate into various phenotypically distinct subsets. Hypoxia enhances the stability of HIF-1α, which in turn promotes metabolic reprogramming and the development of proangiogenic CAFs [42]. Crosstalk with immune and endothelial cells further refines CAF identities, resulting in the development of myofibroblastic (myCAF), inflammatory (iCAF), antigen-presenting (apCAF), vascular, and senescent CAFs, each of which contributes distinctly to tumor progression or, in certain situations, restraint [14, 20, 59, 60].
- **V. Modulators-reinforcing and counter-regulatory influences:** Additional pathways serve as modulators in the transition from fibroblasts to CAFs. Hedgehog signaling facilitates desmoplasia and the proliferation of CAFs, especially in the context of pancreatic cancer [64]. Factors derived from the immune system, including IFN-γ, TNF-α, and IL-1β, enhance the activation of inflammatory CAF [68, 73]. Conversely, the pathways involving Vitamin D receptor (VDR) signaling and retinoic acid can facilitate the reprogramming of CAFs into a quiescent state, thus creating opportunities for therapeutic strategies [25, 26]. Ultimately, cellular senescence plays a role in the heterogeneity of CAFs via the senescence-associated secretory phenotype (SASP), which releases IL-6, IL-8, and matrix metalloproteinases (MMPs) to enhance inflammatory and tumor-promoting activities [59].

Together, these interconnected signaling events demonstrate that fibroblast-to-CAF transition is a multi-layered and dynamic process. Cytokines commence activation, growth factors intensify responses, epigenetic mechanisms and ECM feedback maintain identity, environmental stressors generate diverse phenotypes, and modulatory circuits adjust or counteract activation. This mechanistic framework elucidates the reasons behind the persistence of CAFs within tumors long after the initiating signals have diminished, underscoring the difficulties faced by therapeutic strategies that seek to eliminate, reprogram, or selectively target subsets of CAFs due to their inherent plasticity and functions that depend on context.

#### Mechanisms of CAF-Mediated tumor progression

Building on their diverse origins, subtypes, and spatial niches described in Sect. 2, CAFs have a significant functional impact on tumor biology. Rather than acting as mere passive stromal bystanders, they actively reshape the extracellular matrix, modulate essential signaling pathways, influence the immune environment, and coordinate angiogenesis, invasion, and metastasis.

**Fig. 6:**
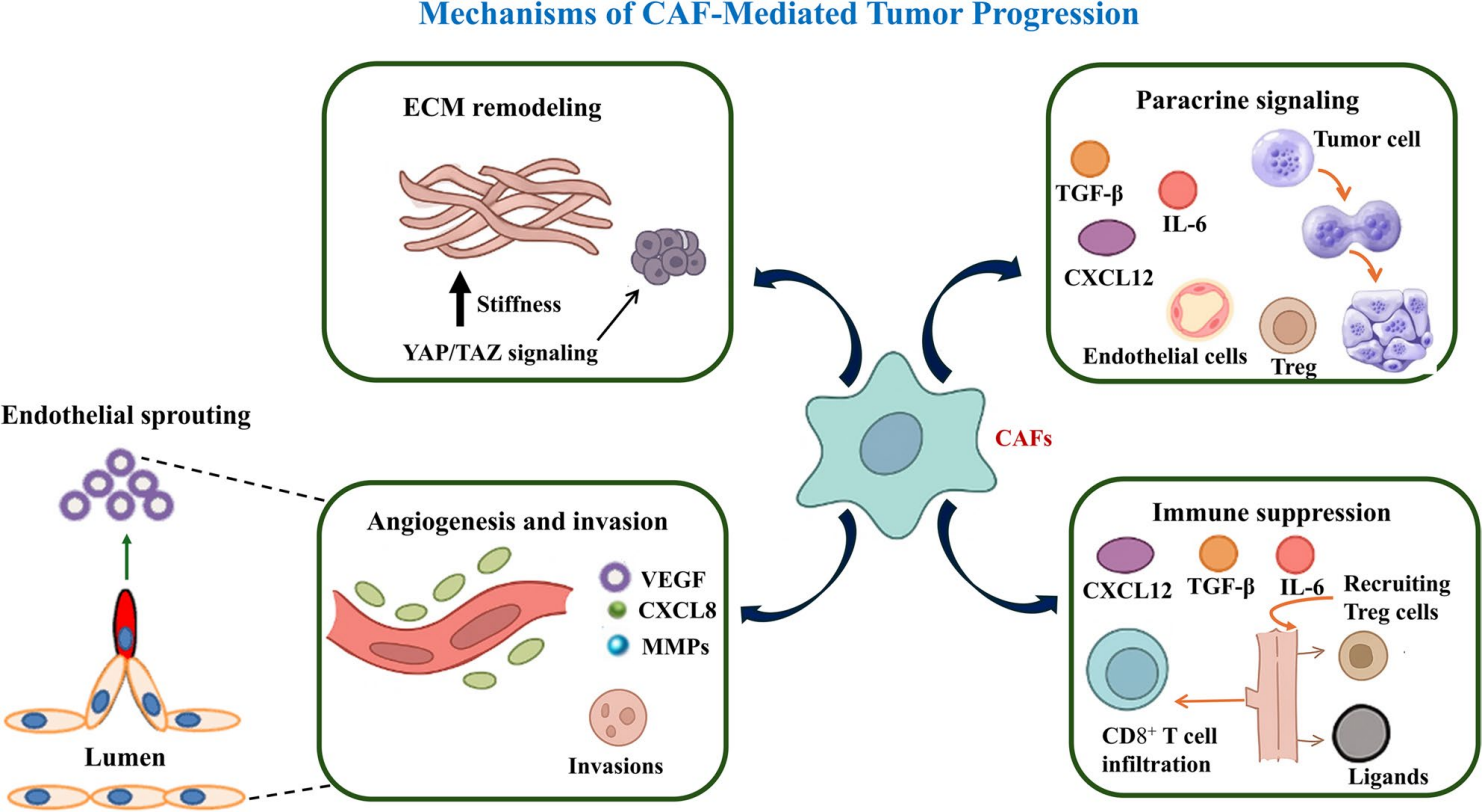
Mechanisms of CAF-mediated tumor progression. CAFs act as central hubs orchestrate multiple tumor-promoting mechanisms including: (1) ECM remodeling: Depositing fibrillar collagens and fibronectin, increasing matrix stiffness and activating YAP/TAZ mechanotransduction [3, 39, 40]. (2) Paracrine signaling: Secreting TGF-β, IL-6, and CXCL12 to drive fibroblast activation, tumor proliferation, and cross-talk with immune and endothelial cells [16, 38, 43]. (3) Angiogenesis & invasion: CAFs promote the formation of new blood vessels (neovascularization) by secreting growth factors like VEGF, which binds to endothelial cells, triggering their invasion into the surrounding matrix, proliferation, and migration towards the stimulus, ultimately leading to the sprouting of new vessels. Additionally, CAFs secrete CXCL8 and MMPs, facilitating ECM degradation and creating invasion tracks for tumor migration, thereby supporting tumor growth and progression [42, 60, 70]. (4) Immune suppression: Excluding CD8⁺ T cells from tumor nests and recruiting regulatory T cells (Tregs) via CXCL12, TGF-β, and checkpoint ligands, thereby fostering an immunosuppressive microenvironment [18, 23, 43]

These mechanisms, although often promoting tumors, can display a duality that is dependent on context, influencing disease trajectories in complex ways. The subsections below outline the primary mechanistic axes through which CAFs drive cancer progression, as depicted in Fig. 5 and 6.

### ECM remodeling and fibrosis

ECM remodeling and fibrosis are defining hallmarks of CAF activity across tumors. CAFs contribute to the deposition of fibrillar collagens, fibronectin, and tenascin C, thereby strengthening a desmoplastic stroma that both aids and limits tumor cells [5, 12]. Fibrosis varies among different types of cancers: pancreatic ductal adenocarcinoma (PDAC) forms extensive collagen-rich capsules that limit perfusion, while the stroma in breast cancer frequently exhibits more irregular fibrotic niches that prevent the infiltration of immune cells [64, 74]. In contrast, tumors with low stroma, like melanoma, show reduced desmoplasia yet continue to depend on CAF-mediated remodeling at their invasive fronts [63].

LOX (Lysyl oxidase) facilitates collagen crosslinking, which stiffens the matrix and triggers the activation of integrin–FAK and YAP/TAZ mechanotransduction pathways. This process strengthens the contractility of CAFs and ensures their continued activation [37]. At the same time, MMPs break down the ECM, which releases bioactive fragments known as matrikines that encourage angiogenesis and invasion [75]. This interaction demonstrates the dual nature of fibrosis: the stroma has the capacity to both encapsulate and limit tumor cells, while simultaneously promoting therapy resistance and hypoxia-induced aggressiveness [18, 19]. Recent spatial multi-omics research indicates that organ-specific ECM niches correlate with clinical outcomes, establishing fibrosis as both a prognostic biomarker and a therapeutic target [15].

Despite progress in understanding CAF-ECM interactions, significant gaps still exist. For example, the exact functions of LOX-mediated collagen crosslinking and other enzymes that modify the matrix in the stiffening driven by CAFs are not fully comprehended [46, 47, 65]. A key unresolved challenge lies in whether the selective targeting of these enzymes can achieve normalization of the tumor stroma without compromising physiological tissue repair, a question that holds significant implications for the safe translation of therapeutic approaches [5, 12].

### Signaling pathways in CAF function

CAF specialization emerges from an interconnected web of growth factor and cytokine signaling. TGF-β/SMAD signaling drives myofibroblastic differentiation, ECM deposition, and immune-suppressive programming [38, 41]. IL-6/STAT3 signaling reinforces inflammatory CAF states, promoting chemoresistance and EMT [16, 55], while NF-κB activation sustains chronic inflammation [43].

These pathways are rarely independent. TGF-β works alongside hypoxia to stabilize myCAF programs, while IL-6/STAT3 cooperates with NF-κB to boost cytokine release in inflammatory CAFs [24]. PDGF and FGF promote the development of proliferative and angiogenic CAF phenotypes [42], whereas the Hedgehog and Wnt pathways govern fibroblast plasticity and sensitivity to therapy [64]. Newly identified regulators encompass AKT3 in head and neck cancers [76] and FGFR2–HIF1α loops that support pro-metastatic CAF programs in ovarian cancer [42, 55]. Epigenetic remodeling also enhances the stability of these states: histone acetylation and DNA methylation establish a “memory” of activation that endures, even after the withdrawal of tumor-derived signals [39].

### Immune modulation and immunosuppression

A primary role of CAFs is to modulate immune responses, functioning as stromal gatekeepers that inhibit anti-tumor immunity and promote immune evasion. This modulation occurs through a blend of physical exclusion, signaling via soluble factors, expression of checkpoints, and interaction with various immune cell lineages.

#### Physical exclusion of effector T cells

 CAFs generate CXCL12, which binds to CXCR4 on CD8⁺ T cells, sequestering them in stromal areas and preventing their infiltration into the tumor [77, 78]. Additionally, the accumulation of ECM creates a physical obstruction that diminishes the ability of T-cells to access the area and restricts the penetration of drugs [73, 79].

#### Soluble immunosuppressive mediators

 Through secretion of TGF-β, IL-6, and prostaglandin E₂, CAFs alter the immune equilibrium by promoting M2 polarization in macrophages, increasing the number of regulatory T cells (Tregs), and diminishing the cytotoxicity of NK cells [68, 71, 80]. The IL-6/STAT3 signaling pathway further strengthens inflammatory CAFs states, which contribute to the persistence of chronic immunosuppression [16].

#### Checkpoint ligand expression

 Apart from cytokines, CAFs can directly inhibit effector cells by expressing checkpoint molecules. Studies have shown that the expression of PD-L1, CD73, and IL-27 on CAFs can suppress the activity of CD8⁺ T-cells and reduce their effectiveness in responding to immune checkpoint blockade [71, 81, 82]. This positions CAFs as active participants in adaptive immune resistance, not simply indirect modulators.

#### Suppression of innate and antigen-presenting cells

 CAFs hinder the maturation of dendritic cells and the presentation of antigens through the secretion of IL-6, VEGF, and TGF-β, consequently restricting the effective priming of T cells [68, 83]. In parallel, CAF-derived factors reduce NK cell infiltration and cytotoxicity, thereby further undermining innate surveillance [71, 79].

#### Subtype-specific contributions

The heterogeneity of CAFs further enhances immune interactions. Inflammatory CAFs (iCAFs) release elevated amounts of IL-6 and CXCL12, which intensifies immune exclusion and promotes Treg recruitment [20, 43]. Antigen-presenting CAFs (apCAFs) express MHC-II and co-stimulatory molecules, resulting in paradoxical effects: they can activate CD4⁺ T cells in some situations while fostering tolerance in different contexts [23]. Simultaneously, myofibroblastic CAFs (myCAFs) are key contributors to immunosuppression, primarily through the indirect mechanisms of dense ECM deposition and TGF-β production [18].

#### Integrative view

By combining physical exclusion, soluble factor signaling, and checkpoint expression, CAFs act as stromal immune checkpoints that manage multi-lineage suppression across T cells, NK cells, macrophages, and dendritic cells. Significantly, these mechanisms play a direct role in conferring resistance to immunotherapies, emphasizing CAFs as both physical and functional obstacles to sustained anti-tumor immunity.

Notably, the CAF-derived signals that reduce immunity, such as VEGF, TGF-β, and matrix-remodeling enzymes, simultaneously promote angiogenesis, invasion, and metastatic dissemination, illustrating the connection between immune evasion and tumor progression mechanisms that will be addressed in the subsequent sections.

### Angiogenesis, Invasion, and metastasis

Furthermore, beyond their involvement in immunosuppression, CAFs are instrumental in facilitating angiogenesis and metastatic spread via synchronized signaling, ECM remodeling, and paracrine interactions with endothelial and cancer cells. This highlights the role of CAFs as dynamic architects of the stroma, integrating vascular remodeling with invasive expansion.

#### Angiogenesis and vascular remodeling

CAFs release a variety of angiogenic mediators, such as VEGF-A, PDGF-C, angiopoietin-1/2, and CXCL8/IL-8, which together promote endothelial sprouting and vascular remodeling [42, 60]. Beyond the role of soluble mediators, CAFs reshape the ECM to release matrix-bound VEGF and construct pro-angiogenic scaffolds. Perivascular CAFs often perform pericyte-like functions, stabilizing newly formed but leaky vessels, thereby ensuring the continuation of abnormal tumor perfusion [71]. The functional duality is apparent: although excessive activity of CAFs leads to abnormal blood vessel formation that contributes to hypoxia and metastasis, some subsets of CAFs can temporarily normalize these vessels, thereby improving the delivery of therapies [64].

#### Lymphangiogenesis

 CAF-derived VEGF-C and VEGF-D promote the proliferation and sprouting of lymphatic endothelial cells, playing a significant role in lymphatic remodeling and nodal metastasis. Additionally, ANGPT2 released by CAFs further destabilizes lymphatic vessels, thereby enhancing the process of metastatic seeding [71]. Clinically, tumors characterized by CAF-rich perivascular niches demonstrate increased rates of nodal involvement and a worse prognosis.

#### Invasion

 CAFs play the role of “leader cells” in the collective migration of cancer cells. They generate aligned collagen and fibronectin fibers, which serve as invasion highways, through Rho/ROCK-driven contractility, integrin α11, and podoplanin [7, 54]. By secreting LOX and MMPs, CAFs further alter the matrix to create pathways for migration. CAFs also offer directional signals through HGF, CXCL12, and periostin, thereby strengthening invasive routes [84].

**Metastasis and Pre-metastatic niche formation:** CAF-derived exosomes are loaded with pro-metastatic cargo:

a) miR-21 enhances fibroblast activation and EMT programs.

b) Fibronectin and LOX stiffen pre-metastatic niches in lung and liver.

c) TGF-β and MMPs remodel extracellular niches and suppress immune surveillance.

 These vesicles attract myeloid cells derived from bone marrow and modify vascular permeability, preparing distant organs for colonization [15]. CAF-platelet interactions provide protection to circulating tumor cells, thereby improving their survival in the bloodstream. Organ-specific metastasis is shaped by the activities of CAFs: lung metastases are connected to fibronectin-rich niches, while liver metastases are advanced by the stiffening of the stroma that is driven by CAFs [58]. Overall, the contributions of CAF-driven angiogenesis, invasion, and metastasis to cancer dissemination are multifaceted. While most of their effects are conducive to tumor advancement, there are dual roles where CAF-mediated vessel normalization or stromal encapsulation can momentarily hinder progression, illustrating the complexity of their roles. Beyond their mechanical and biochemical signaling functions, exosomes produced by CAFs have been recognized as key players in the communication between tumors and the surrounding stroma, extending the reach of fibroblast influence beyond local paracrine signaling [8, 9, 50]. These vesicles contain a diverse array of molecules that facilitate tumor progression and metastasis by reprogramming the transcriptional, metabolic, and structural characteristics of both cancer and stromal cells. The subsequent detailed summary elucidates the mechanistic and translational consequences of CAF exosomes: (i) miRNAs like miR-21, miR-92a, and miR-181d facilitate epithelial-mesenchymal transition (EMT), chemoresistance, and stem-like characteristics [9, 32, 50]; (ii) lncRNAs such as H19 and PVT1 stimulate angiogenesis and preserve cancer stemness [32, 50]; (iii) proteins including lysyl oxidase (LOX), fibronectin, TGF-β, MMP-2/9, and integrins αvβ3/α6β4 modify the extracellular matrix (ECM) and create pre-metastatic niches through stromal stiffening and recruitment of myeloid cells [8, 9, 41]; and (iv) metabolic enzymes like PKM2 and GLS1 enhance redox adaptation and metabolic interdependence between CAFs and cancer cells [32, 45, 50]. Together, these exosomal cargos combine structural remodeling with metabolic resilience, allowing tumors to metastasize and withstand therapy. From a mechanistic perspective, this supports the notion that CAF exosomes serve as systemic agents of microenvironmental conditioning and may act as potential circulating biomarkers or therapeutic targets in precision stromal oncology [8, 32, 36, 50]. 

### Therapy resistance

CAF-mediated resistance to therapy originates from various physical, biochemical, immune, and metabolic factors. The dense fibrotic extracellular matrix (ECM) obstructs drug penetration, whereas integrin-FAK signaling transmits survival signals during chemotherapy [37, 85]. IL-6, CXCL12, and IGFBP7 engage the STAT3 and AKT pathways, leading to a decrease in cytotoxicity [16, 56]. CAFs play a role in the resistance to immune checkpoint blockade by preventing the infiltration of CD8 + T cells and maintaining niches rich in Treg cells [78].

From a metabolic perspective, CAFs participate in a phenomenon known as the “reverse Warburg effect,” wherein they generate lactate and ketone bodies that serve as energy sources for oxidative tumor metabolism [24, 45]. Functional duality is also noted: by changing metabolic dependencies, CAFs may ironically make tumors more susceptible to metabolic inhibitors, which suggests new therapeutic possibilities.

Furthermore, radiotherapy solidifies the immunosuppressive traits of CAFs, allowing irradiated CAFs to uphold MDSC recruitment and checkpoint expression [86]. Clinical transcriptomic analysis demonstrates that CAF signatures consistently serve as predictors of inadequate therapeutic responses across multiple cancers [58].

Beyond their metabolic functions, CAFs have surfaced as essential regulators of ferroptosis, a type of cell death that relies on iron and is driven by lipid peroxidation, thereby influencing the response of tumors to treatment [87]. CAFs act to suppress ferroptosis in surrounding tumor cells by employing integrated mechanisms related to redox, lipid, vesicular, and immune functions. By exporting glutathione (GSH) and cystine through the SLC7A11/xCT transporter, CAFs effectively restore intracellular thiol pools and uphold redox stability in cancer cells [88]. They also affect lipid composition through the secretion of lipids and enzymes that regulate ACSL4, which limits the phospholipid peroxidation essential for ferroptotic death [89]. CAF-derived exosomes transport GPX4-stabilizing proteins, miR-522, and long-chain fatty acids, increasing antioxidant capacity and directly suppressing ferroptotic pathways [90, 91]. Moreover, the role of CAFs in sustaining an antioxidant-rich microenvironment alleviates T cell-induced oxidative stress, consequently impairing the effectiveness of immunotherapy [92]. In support of these findings, Ebrahimnezhad et al. revealed that CAF-mediated ferroptosis resistance is a crucial element in adaptive immune evasion and the shortcomings of checkpoint blockade [93]. As a result, targeting the CAF redox axis by inhibiting SLC7A11, GPX4, or lipid-remodeling enzymes might reinstate sensitivity to ferroptosis and strengthen immunotherapy [89–91, 93].

### Integrative perspectives

CAFs integrate structural remodeling, paracrine signaling, immune modulation, and vascular regulation into a unified stromal program that supports tumor progression [3, 5]. Recent discoveries have revealed several validated principles that go beyond mere descriptive mechanisms:

**Spatial organization as a determinant of outcome:** Perivascular, invasive, and desmoplastic CAF niches hold significant prognostic value, with perivascular CAFs associated with angiogenesis and invasive-front CAFs related to collective migration [15, 23, 58].

**The ECM as scaffold and signal reservoir: **The process of fibrotic remodeling not only provides structural support but also liberates latent growth factors, including VEGF and TGF-β, which connect biomechanics to pro-tumorigenic signaling [12, 13, 37].

**Reinforcing signaling loops sustain CAF activation**: Crosstalk between the TGF-β/SMAD, HIF-1α, and IL-6/STAT3–NF-κB signaling pathways stabilizes CAF phenotypes and heightens resistance to therapy [38, 42, 68].

#### Epigenetic scarring preserves stromal memory

 The processes of histone acetylation and DNA methylation “lock in” the activation of CAFs, which remains evident even after tumor-derived signals have been withdrawn, a phenomenon that correlates with the persistence of therapy [25, 39].

**Metabolic exchange creates vulnerabilities:** The phenomenon known as the “reverse Warburg effect,” in which CAFs release lactate and alanine, facilitates tumor metabolism while revealing reliance on transporters and metabolic enzymes [24, 45].

**CAF-mediated immune suppression acts as a stromal checkpoint**: Through CXCL12-mediated T-cell exclusion, alongside TGF-β and IL-6 signaling, and the expression of checkpoint ligands including PD-L1 and CD73, CAFs play the role of immune gatekeepers, regulating the interactions between tumors and the immune response [71, 77, 79].

**Systemic conditioning via exosomes primes metastasis**: Vesicles derived from CAFs, which carry LOX, fibronectin, and miR-21, alter the distant stroma and draw in myeloid progenitors, leading to the formation of pre-metastatic niches [15, 84].

**Functional duality mandates selective targeting:** The protective functions of encapsulating myCAFs stand in stark contrast to the pro-invasive roles of iCAFs and proangiogenic CAFs, highlighting the dangers associated with indiscriminate depletion [18, 19].

#### Integrated biomarkers are essential for translation

 Profiles that integrate CAF subtype markers (Supplementary Table 1), ECM indices, spatial immune exclusion, and metabolic signatures demonstrate superior performance compared to individual metrics in forecasting therapeutic results [15, 58, 71].

Supplementary table 1. CAF subtype markers across various cancers. CAF subtypes exhibit distinct marker profiles, which can vary depending on the cancer type. Identifying these markers is crucial for understanding CAF biology and developing targeted therapies. Understanding these markers can provide following insights into CAF function and cancer progression: 1). CAF heterogeneity and plasticity, 2). Role of CAFs in tumor progression and metastasis, 3). CAF-immune cell interactions, 4). Metabolic reprogramming in CAFs, 5). Therapeutic targeting of CAFs, 6. CAF biomarkers and diagnostics.

Collectively, these principles establish CAFs as proactive architects of tumor ecosystems and systemic regulators of progression. Their capacity to concurrently remodel tissue architecture, inhibit immunity, and prepare distant sites highlights the necessity for targeted, context-specific interventions. The following section explores CAF-targeted therapeutic strategies, translating these mechanistic insights into precision approaches for clinical application.

While the previously discussed mechanisms underscore CAF-driven tumor promotion, a growing collection of evidence shows that CAFs additionally have context-dependent tumor-restraining capabilities, making them a therapeutic double-edged sword. Importantly, the experimental elimination of myofibroblastic CAFs in pancreatic cancer led to a faster disease progression and a decrease in survival, indicating that these stromal cells may have protective roles under certain circumstances [18, 19]. On the other hand, certain CAF subsets or spatially arranged stromal architectures can either limit vascular invasion or bolster anti-tumor immunity when specific conditions are met [15, 23]. Recognizing this duality is vital: it opposes indiscriminate stromal ablation and instead endorses approaches that distinguish tumor-promoting CAFs from tumor-restraining ones or strive to reprogram CAFs instead of just eliminating them.

The cumulative evidence underscores the complex interplay of mechanisms by which cancer-associated fibroblasts (CAFs) regulate tumor progression. A summary of the key CAF-driven processes and their molecular mediators is provided in Table 3.

**Table 3:** Key mechanisms of CAF-mediated tumor progression: summary of major pathways and functional effects of CAFs, including ECM remodeling, signaling cascades, immune modulation, angiogenesis/invasion, and therapy resistance

CAF Subtype	Key Drivers/Pathways	Primary Mechanisms	Clinical Impact	Therapeutic strategies	References
myCAF (myofibroblastic CAFs)	TGF-β/SMAD, YAP/TAZ, ECM stiffness	ECM deposition, fibrosis, mechanotransduction, stromal encapsulation	Promotes therapy resistance via drug exclusion; may restrain early tumor spread	VDR agonists; RA-induced quiescence; stromal normalization	[25, 26, 37, 38, 64]
iCAF (inflammatory CAFs)	IL-6/STAT3, NF-κB, FOS	Cytokine/chemokine secretion (IL-6, CXCL12, CCL2), recruitment of immunosuppressive cells	Immune exclusion, therapy resistance; context-dependent immune activation	IL-6/STAT3 inhibitors; CXCR4 blockade; TGF-βR inhibition	[15, 16, 27, 43, 55]
apCAF (antigen-presenting CAFs)	IFN-γ/JAK-STAT, epigenetic remodeling	MHC-II expression, antigen presentation to CD4 + T cells	Can promote T cell activation but also tolerance	Explored for immunotherapy augmentation	[20]
Metabolic CAFs	HIF-1α, AMPK, lactate shuttle	Reverse Warburg effect, nutrient buffering, metabolite exchange	Fuels tumor metabolism, therapy resistance; may sensitize tumors to metabolic inhibitors	Targeting lactate transporters; metabolic inhibitors	[24, 45]
Senescent CAFs	DNA damage, oxidative stress, SASP	SASP secretion (IL-8, MMPs, CXCLs), remodeling of tumor niche	Promotes chronic inflammation and tumor progression	Senolytic agents; SASP-targeting therapies	[59]
Proangiogenic CAFs	Hypoxia/HIF-1α, PDGF, VEGF	VEGF/PDGF secretion, vessel stabilization, ECM scaffolding	Enhances tumor angiogenesis and metastasis	Anti-angiogenic agents; PDGF inhibitors	[42, 60]
Vascular CAFs	VEGF-C/D, lymphangiogenic signaling	Lymphangiogenesis, lymphatic vessel remodeling	Facilitates nodal metastasis and immune cell trafficking	VEGF-C/D blockade; targeting lymphatic niches	[54, 71]

## Therapeutic interventions targeting CAFs

CAFs have been regarded for a long time as potential therapeutic targets owing to their crucial involvement in the regulation of tumor growth, ECM remodeling, angiogenesis, and immune suppression. Nevertheless, the application of these therapies has faced significant challenges, as CAFs are not a uniform group but are instead highly heterogeneous and contextually dependent. Strategies have consequently progressed from unselective depletion to targeted inhibition of specific pathways and functional reprogramming. This section delineates the primary therapeutic modalities, their underlying mechanisms, and significant limitations.

### Targeting CAF-Specific markers

Fibroblast activation protein (FAP) is recognized as the standard marker for targeting CAFs in therapy. It is a serine protease that is elevated in the tumor stroma, promoting the breakdown of the extracellular matrix (ECM) and facilitating immune suppression. Subsequent strategies utilizing systemic FAP depletion led to improved immune infiltration in preclinical models, yet they also induced cachexia, anemia, and bone marrow toxicity due to the presence of FAP in normal tissues [18, 94]. The preclinical toxicities encountered posed significant obstacles to clinical development. Initial trials involving FAP-directed antibodies and vaccines did not show substantial antitumor efficacy and were discontinued because of dose-limiting toxicities, prompting a strategic pivot towards imaging and theranostic uses of FAP ligands instead of systemic depletion [95, 96]. In a similar manner, α-SMA has been a target; however, its extensive expression in fibroblasts involved in wound healing restricts specificity.

To address these limitations, innovative strategies focus on spatial or tumor-specific targeting. Nanoparticle formulations and antibody–drug conjugates directed by tenascin-C or α-SMA selectively transport cytotoxic drugs to stroma abundant in CAFs [97]. Near-infrared (NIR) photoimmunotherapy utilizing anti-FAP antibodies facilitates targeted stromal ablation while minimizing systemic exposure [94]. Recent advancements in immunotherapy encompass FAP-directed CAR-T cells and bispecific T-cell engagers, which are being developed with logic-gated safety switches and localized delivery mechanisms to minimize on-target/off-tumor toxicity [28, 98]. These enhancements illustrate the transition of CAF marker targeting from general ablation techniques to precision-guided approaches.

### Targeting CAF signaling pathways

Numerous signaling pathways within CAFs have been explored as potential therapeutic targets, encompassing developmental, inflammatory, metabolic, and immunoregulatory mechanisms. Although initial research sparked excitement, subsequent translation has uncovered both therapeutic prospects and inherent pitfalls.

Hedgehog signaling exemplifies a well-established paradox. The genetic and pharmacological removal of Hedgehog-responsive fibroblasts in pancreatic cancer models led to a decrease in stromal content, yet it unexpectedly hastened tumor growth and reduced survival [18, 19]. These outcomes reveal that certain subsets of CAFs perform tumor-restraining functions, and that broad pathway inhibition can be harmful. This contradiction was demonstrated in clinical trials: the Hedgehog inhibitor IPI-926 (saridegib) was prematurely stopped in pancreatic cancer after Phase II trials showed increased disease progression and poorer overall outcomes, highlighting the risks of stromal depletion in patients [64, 99].

FAP-targeted interventions further demonstrate these risks. While monoclonal antibodies, vaccines, and CAR-T cells that are directed against FAP have shown tumor reduction in preclinical models, the systemic depletion of FAP caused serious toxicities, including cachexia-like effects, due to the low-level expression of FAP in normal stromal tissues [96, 98].

TGF-β pathway inhibition has exhibited more sophisticated effects. Small-molecule inhibitors such as galunisertib have been effective in restoring antitumor immunity and in creating a synergistic effect with checkpoint blockade [27]. On the other hand, systemic suppression may jeopardize tissue repair and the stability of immune homeostasis. Correspondingly, the inhibition of PDGF lessened the feedback activation of hypoxia-inducible factors and curtailed the progression of ovarian tumors [42].

Beyond these traditional cascades, oxidative stress pathways have become increasingly important. Research indicates that CAFs facilitate NOX4-dependent ROS production, which drives the exclusion of CD8⁺ T-cells from tumors. The pharmacological inhibition of NOX4 restored T-cell infiltration and enhanced the effectiveness of immunotherapy, highlighting the crucial role of redox signaling in immune evasion orchestrated by CAFs [78].

A further axis concerns fibroblast growth factor signaling. Another investigation indicated that inflammatory CAFs, utilizing the SFRP1-FGFR2-HIF1α axis, drive hypoxia-induced metastasis in colorectal cancer liver colonization. This underscores that CAF signaling is not solely tumor-promoting in a localized manner but is also essential in the establishment of metastatic niches [55].

Ultimately, engineered constructs demonstrate the application of CAF signaling biology in therapeutic advancements. The bispecific fusion protein M7824 (anti–PD-L1/TGFβR2) concurrently inhibits CAF-derived TGF-β signaling and tumor PD-L1 checkpoint pathways, resulting in enhanced antitumor responses in preclinical models [100]. By merging CAF-targeted inhibition with immune checkpoint blockade, these agents effectively overcome the drawbacks of single-pathway suppression.

Collectively, the evidence from Hedgehog, FAP, oxidative stress, growth factor, and bispecific targeting methods indicates that CAF signaling is both heterogeneous and context-dependent. The failures of trials underscore the risks associated with indiscriminate inhibition, whereas the development of selective and combinatorial strategies illustrates how analyzing CAF subtypes and their pathways can enhance therapeutic targeting.

### Reprogramming CAF signalling pathway

An alternative to depletion or direct inhibition is CAF reprogramming, which aims to restore quiescent or tumor-restraining functions while maintaining stromal architecture. This approach leverages the plasticity of fibroblasts and their ability to shift between active and inactive states.

Pharmacological interventions serve as a prime example of this strategy. Vitamin D receptor (VDR) agonists have been demonstrated to transform pancreatic stellate cells into a quiescent phenotype, thereby decreasing desmoplasia and enhancing the delivery of chemotherapy [25]. Similarly, all-trans retinoic acid (ATRA) prompted quiescence in pancreatic cancer-associated fibroblasts (CAFs), reducing paracrine Wnt–β-catenin signaling and hindering tumor advancement. Both strategies illustrate how reprogramming mitigates the harmful consequences linked to CAF depletion.

Epigenetic remodeling provides an additional layer of CAFs reprogramming. Histone acetylation and DNA methylation preserve an “activation memory” that upholds the identity of CAFs, even in the absence of tumor-derived signals. The inhibition of these processes may lead to a stabilization of the reversal of the CAF phenotype. This emphasizes that reprogramming can counteract pro-invasive signaling instead of completely removing fibroblasts. Recently, metabolic rewiring has been identified as a potential therapeutic target: Cancer-associated fibroblasts (CAFs) support tumors through lactate shuttling and lipid biosynthesis. Inhibitors that target lactate transport and lipid metabolism can interrupt these paracrine loops, presenting a novel therapeutic approach [24, 45].

Apart from pharmacology, the role of immune crosstalk in the transitions of CAF states is significant. For example, it has been shown that T-cell–driven signaling feedback can influence the expression of inhibitory ligands, including CD73 and IL-27, thus altering their impact on the tumor immune microenvironment [81]. These findings reinforce that CAF phenotypes are not static but rather dynamic, shaped by their immune and stromal environment.

Collectively, reprogramming methods illustrate the possibility of altering CAF functions instead of eliminating them. This approach offers a mechanistic rationale for the observation that depletion may hasten disease progression, while reprogramming maintains stromal integrity and diminishes tumor-promoting signals.

#### Challenges in CAF reprogramming

 The reprogramming of CAFs poses significant biological and therapeutic challenges. The stability of the reprogrammed state is still uncertain, as re-exposure to cytokines derived from the TME (such as IL-1β, TGF-β, and hypoxia) can lead to swift reactivation and a reversion to a pro-tumorigenic phenotype [39, 81]. The maintenance of epigenetic memory through sustained histone acetylation and DNA methylation may hinder full phenotypic reversal [39]. Metabolic stress or oxidative signals induced by therapy can awaken dormant fibroblasts, which may lead to tumor recurrence [25, 26]. Another issue is that extensive reprogramming might unintentionally hinder fibroblast-driven tissue repair or immune monitoring, resulting in stromal vulnerability [81].

#### Limitations and potential solutions

 To overcome these limitations, it is important to adopt context-specific modulation that integrates transient reprogramming agents with immune checkpoint or metabolic therapies, thereby bolstering the stability of anti-tumor fibroblast states [25, 26, 81]. Recent research emphasizes the temporary characteristics of reprogrammed states. Additionally, recent in vivo studies involving pancreatic ductal adenocarcinoma (PDAC) models have indicated a return of CAF after stopping VDR agonists, which highlights the transient qualities of these reprogrammed states [25]. Likewise, quiescence that is induced by VDR or ATRA might be reversible through epigenetic processes, with reactivation happening because of continuous TGF-β, IL-1, or mechanical stress [26, 39]. This underscores the necessity of creating robust yet reversible reprogramming frameworks, where stromal normalization is preserved without compromising the physiological functions of fibroblasts. Moreover, enduring metabolic shifts or lasting epigenetic changes could jeopardize the standard balance of fibroblasts, underscoring the significance of interventions that are both contextually relevant and temporally regulated [25, 26, 81].

Together, these insights offer a well-rounded and realistic evaluation of the potential and limitations of CAF reprogramming as a therapeutic option [25, 26, 39, 81].

### CAF-Immune crosstalk targeting

CAFs have a substantial effect on immune evasion through their involvement in chemokine signaling and matrix barriers. One of the primary mechanisms is the CXCL12-CXCR4 axis, in which CAF-derived CXCL12 generates both a physical and chemotactic barrier to the infiltration of cytotoxic T-cells. Inhibiting CXCR4 allows CD8 + T-cells to access tumors more effectively and improves responses to checkpoint blockade [101, 102].

Additional immunomodulatory pathways encompass the FAP-STAT3-CCL2 circuit, which attracts myeloid-derived suppressor cells and fosters an immunosuppressive microenvironment; inhibiting this pathway diminishes MDSC infiltration and reinstates anti-tumor immunity [44]. Significantly, NOX4 inhibitors connect ECM remodeling with immune modulation, concurrently decreasing matrix stiffness while enhancing T-cell infiltration [78]. Collectively, these insights illustrate that targeting immune-crosstalk is among the most dynamic and translationally pertinent strategies directed at CAFs.

### Current state of research

Various CAF-targeting therapies are moving forward in both preclinical and clinical testing phases. FAP-targeted CAR-T therapies, as well as bispecific antibodies that link CAF antigens to immune activation, are being investigated in early-phase trials [28, 100]. Agents that aim to target both CAFs and immune checkpoints at the same time, such as PD-L1/TGF-β bispecific antibodies, have revealed significant activity in tumors that are resistant to single-agent therapy [103]. Clinical trials are assessing the effects of vitamin D analogs on stromal reprogramming in pancreatic cancer, with preliminary results indicating changes in stromal structure and better responsiveness to treatment [25, 104]. These studies emphasize the translational progress in therapies directed at CAF, although extensive validation is still awaited.

From a translational viewpoint, the clinical path of CAF-targeting highlights both potential and difficulties. Initial excitement regarding FAP-targeted depletion strategies was moderated by systemic toxicities and unexpected stromal effects [18, 19]. Recent advancements emphasize the promise of utilizing biomarkers for patient selection and theranostic strategies. FAP-based PET tracers now facilitate noninvasive measurement of stromal burden, serving as a companion diagnostic to categorize patients for CAF-modulating treatments [96]. Correspondingly, CD10⁺GPR77⁺ CAFs, strongly connected to cancer stemness and chemoresistance, might characterize a particular group of patients who are most likely to receive benefits from CAF-focused treatments [34] [Su et al., 2018]. Current trials are progressively incorporating these biomarkers, marking a significant advancement in the field of precision stromal oncology.

A significant conceptual development arising from these therapeutic approaches is the differentiation between CAF depletion and CAF reprogramming. Initial attempts primarily concentrated on the depletion of CAFs using markers like FAP or through pathway inhibition; however, preclinical findings revealed that indiscriminate ablation of CAFs can unexpectedly hasten tumor growth and negatively affect survival [18, 19]. This understanding has redirected focus towards reprogramming strategies, in which agents like vitamin D analogs, retinoids, or TGF-β modulators transform CAFs into more quiescent or tumor-restraining phenotypes [15, 25, 26]. Framing current interventions within this depletion versus reprogramming, we gain a unified perspective that more precisely mirrors the variability and flexibility of CAFs, while also furnishing a more sophisticated strategy for clinical translation as specified in the Table 4.

**Table 4:** Selected clinical trials of CAF-targeted or stromal-modulating therapies

Target/Strategy	Agent/Approach	Cancer Type(s)	Trial ID(s)	Clinical Outcome/Status	References
FAP-targeted antibody	Sibrotuzumab (anti-FAP mAb)	Colorectal, NSCLC	(Phase I/II, discontinued)	Safe, but limited efficacy; program halted	[95, 105]
FAP-targeted CAR-T	FAP-CAR-T cells	Solid tumors	NCT01722149, NCT03932565	Early safety established; efficacy under investigation	[106, 107]
FAP theranostics	FAPI-PET tracers, radioligands	Multiple solid tumors	NCT04459273, NCT05263700	High imaging sensitivity; therapeutic evaluation ongoing	[108, 109], 110– [111]
TGF-β inhibitor	Galunisertib (LY2157299)	Pancreatic, HCC, others	NCT01373164	Improved immune activation; modest survival gains	[112–114]
Bispecific PD-L1/TGF-β trap	Bintrafusp alfa (M7824)	NSCLC, HPV+, GI cancers	NCT03631706, NCT02517398	Immune activation; Phase III failed vs. pembrolizumab	[115, 116]
Vitamin D analog	Paricalcitol + Gemcitabine ± Nab-Paclitaxel	Pancreatic cancer	NCT03520790, NCT03519308, NCT02767557	Early-phase; stromal remodeling; drug penetration improved	[25, 117–119]
Retinoid	All-trans retinoic acid (ATRA)	Pancreatic cancer (early clinical)	NCT03999684, NCT02775370	Reprograms CAFs to quiescent phenotype	[26, 120, 121]

However, despite these developments, clinical experience has demonstrated considerable limitations and unexpected failures, which are discussed in (Section Challenges and Limitations).

### Challenges and limitations

Notwithstanding significant advancements, various obstacles hinder the clinical advancement of CAF-targeting therapies. Firstly, the heterogeneity of CAFs complicates the design of therapies: not every subset promotes tumors, and indiscriminate depletion may inadvertently exacerbate outcomes, as evidenced in models of pancreatic cancer [18, 19]. Secondly, the plasticity of CAF facilitates swift adaptation, which may lead to the risks of therapeutic resistance or rebound activation. Thirdly, the absence of markers exclusive to CAF restricts the precision of targeted strategies, whereas the systemic inhibition of central pathways like TGF-β or PDGF entails considerable toxicity [42]. An additional challenge emerges from the constraints of existing in vitro models, which frequently do not accurately mimic the intricate spatial, mechanical, and immunological environment of CAFs in vivo. This inconsistency hinders the precise modeling of CAF-tumor and CAF-immune interactions, limiting the translational significance of numerous preclinical results and adding to the elevated rate of failure in clinical translation [3, 5]. Finally, CAFs are instrumental in the processes of wound healing and homeostasis, which raises concerns that extended suppression could negatively affect normal tissue function.

As specified in (Section Targeting CAF-Specific Markers), FAP-targeted strategies are hindered by toxicity: while preliminary preclinical data showed promise, clinical experiences have demonstrated cachexia and anemia caused by low-level FAP expression in normal tissues [95]. Novel techniques such as radionuclide-based theranostic tracers [96], photoimmunotherapy [94], and FAP-targeted CAR-T cells [28] indicate improved accuracy, yet they are still in the preliminary phases of translation. Likewise, as elaborated in (Section Targeting CAF Signaling Pathways), the suppression of the Hedgehog pathway initially resulted in a reduction of stromal content in pancreatic cancer, but paradoxically hastened tumor progression in clinical trials, illustrating the context-dependent nature of CAFs functions [18, 19, 64].

These lessons stress that CAFs are not uniformly pathogenic and that therapeutic strategies must recognize their dual, at times tumor-restraining, functions. As elaborated in (Section Translational outlook), overcoming these barriers will demand a shift from indiscriminate depletion to biomarker-guided reprogramming and rational combinatorial approaches. Progress in spatial multi-omics, molecular profiling, and adaptive trial design is starting to facilitate this transition, establishing the groundwork for a new age of precision stromal oncology [15, 82].

## Translational outlook

As CAF-targeted treatments develop, the task of translating these innovations into effective clinical interventions remains a critical challenge. Preliminary trial experiences have revealed the possibilities and limitations of stromal targeting, shaping a future path where CAF modulation is improved through precision methodologies, strategic combinations, and tailored medicine.

### CAF-Targeting in clinical trials: lessons learned and ongoing efforts

Despite the promising findings from preclinical studies regarding CAFs as targets for therapy, the process of translating these findings into clinical practice has been challenging. The initial optimism has been dampened by inconsistent responses, context-sensitive effects, and concerns regarding safety. Moreover, an increasing number of clinical trials is actively researching CAF-targeted interventions across multiple cancer types, leading to both enlightening failures and optimistic developments.

#### FAP-Directed approaches

FAP remains the leading CAF marker in clinical advancement. Antibody-based methods, including bispecific constructs such as RG7386 (FAP-DR5), have demonstrated targeted stromal engagement and tumor apoptosis in preclinical trials; nevertheless, the translation to clinical use has been limited due to modest efficacy and safety worries concerning off-tumor FAP expression [122]. Recent advancements encompass near-infrared photoimmunotherapy utilizing humanized anti-FAP antibodies in patient-derived xenografts, which resulted in significant stromal ablation accompanied by tumor regression, indicating a resurgence of clinical potential [94]. In addition, radionuclide-based FAP ligands are being investigated for their potential in imaging and therapy, thus presenting theranostic benefits [96].

#### Hedgehog and TGF-β pathway Inhibition

Given the significance of CAFs in paracrine signaling, there has been considerable exploration into pathway inhibition. Although Hedgehog inhibition initially showed potential in preclinical models of pancreatic cancer, later clinical trials indicated unexpected outcomes, including a rise in tumor aggressiveness tied to stromal depletion [64]. Correspondingly, targeting TGF-β with agents like galunisertib has resulted in prolonged immune activation and tumor regression in particular subsets of patients, especially when combined with checkpoint inhibitors; however, the responses observed with monotherapy were constrained [27]. These experiences illustrate the critical need for exact patient stratification and rational combinations. For instance, the clinical assessment of the Hedgehog inhibitor IPI-926 in pancreatic cancer was concluded prematurely because of accelerated disease progression [64], whereas galunisertib in Phase II trials provided only limited survival advantages, emphasizing the challenges of converting stromal biology into lasting clinical results.

#### Stromal reprogramming via nuclear receptors

Instead of eradicating CAFs, reprogramming techniques aim to return them to a quiescent phenotype. The Vitamin D receptor (VDR) agonist calcipotriol has demonstrated its ability to remodel pancreatic stroma and boost chemotherapy responses [25]. In pancreatic cancer models, retinoic acid induced quiescence in stellate cells and suppressed Wnt signaling that promotes tumors [26]. While largely at early clinical exploration, these interventions demonstrate a significant shift towards the normalization of stromal tissue.

#### Immunotherapy-oriented CAF modulation

The immunosuppressive capacity of CAFs has inspired trials that merge CAF modulation with immunotherapeutic approaches. Such strategies include bispecific constructs that address both PD-L1 and TGF-β signaling (M7824), which have shown to bolster anti-tumor immunity in preliminary studies [100]. More recently, CAR-T cells that have been engineered to target both tumor cells and FAP-expressing stroma effectively have achieved notable preclinical success in pancreatic cancer, advancing towards translational development [28]. These designs illustrate how interventions directed by CAF can work in synergy with therapies based on the immune system.

A significant lesson derived from these trials is the importance of biomarkers that can differentiate patients by their stromal context. Innovative methods encompass FAP-based PET tracers for non-invasive imaging of CAF [96], transcriptional signatures derived from CAF associated with immune exclusion [82], and patterns of spatial CAF heterogeneity revealed through single-cell and multi-omics profiling [15]. The integration of biomarkers as stratification instruments and trial endpoints will be imperative for pinpointing patients who are most likely to derive benefits from CAF-modulating therapies.

Overall, the clinical journey of CAF-targeting emphasizes the risks of indiscriminate stromal depletion and the hopeful prospects of selective, combinatorial, and reprogramming-based methodologies. Despite positive results from various strategies, the application in clinical settings has been uneven. A primary reason is the dependence on model organisms, which, despite being vital for mechanistic studies, fail to fully emulate the heterogeneity, plasticity, and stromal-immune interactions found in human CAFs [123]. The knowledge acquired points to a future where therapies aimed at CAF are blended into multimodal approaches that are specifically tailored to the heterogeneity of CAF and the type of cancer involved. Apart from direct stromal targeting, a key translational opportunity involves merging CAF modulation with immuno-oncology, where the stromal barrier is instrumental in determining the immune response.

#### Integrating CAF modulation with Immuno-Oncology

One of the critical translational challenges involves the synchronization of CAF-directed strategies with immuno-oncology. CAFs are being increasingly identified as vital mediators of immune exclusion, establishing physical barriers around tumor nests and secreting immunosuppressive cytokines that diminish the infiltration and effectiveness of effector T-cells [68, 73]. Mechanistic insights reveal that different subsets of CAFs regulate immune dynamics in distinct ways; myofibroblastic CAFs frequently enhance T-cell exclusion, whereas inflammatory CAFs may induce exhaustion phenotypes due to continuous cytokine exposure [23, 58].

These findings support the rationale for combinatorial strategies. CAF-reprogramming approaches (like VDR or retinoid agonists) could be integrated with checkpoint blockade to eliminate physical barriers and encourage immune infiltration. Simultaneous strategies harness engineered cellular therapies: CAR-T or TCR-T cells, which are co-armed with anti-FAP capabilities, are being developed to directly transform the stroma while ensuring tumor cytotoxicity [28, 98]. Oncolytic virotherapy introduces an additional dimension, utilizing CAF-targeting viral vectors designed to release FAP bispecific engagers that concurrently reduce stroma and stimulate T cells [72].

The challenge of translation involves finding a balance between efficacy and safety. CAF depletion could potentially worsen tumor aggression in specific situations, while excessive immune stimulation increases the likelihood of autoimmunity. Nonetheless, the greatest potential resides in thoughtfully designed combinations, where CAF modulation serves as a “microenvironmental primer” to fully harness the capabilities of immunotherapies. The diverse outcomes observed in clinical trials further illustrate that not all patients gain equal advantages from CAF-targeted methods, emphasizing the pressing requirement for biomarkers to facilitate patient stratification.

#### Biomarkers and patient stratification

The diversity and plasticity of CAFs highlight the need for biomarkers to guide patient selection and therapeutic monitoring. Existing clinical strategies typically consider CAFs as a uniform target, which has likely contributed to the failure of trials. Subtype-specific markers including FAP, α-SMA, tenascin-C, and cytokine secretion patterns reflect certain aspects of this heterogeneity [6, 11]. Notably, CD10⁺GPR77⁺ CAFs have been associated with cancer stemness and chemoresistance, making them significant targets [34]. From a translational perspective, these subset-specific markers may also act as stratification tools in adaptive trials. For instance, CD10⁺GPR77⁺ CAFs could pinpoint patients exhibiting stemness-driven chemoresistance who qualify for combination therapies [34]. Meanwhile, IL-6–high CAFs, which have already been linked to resistance phenotypes, might function as predictive biomarkers for trials that include IL-6 blockade or strategies for stromal reprogramming [97]. Incorporating these stromal markers as eligibility criteria or secondary endpoints would enable CAF biology to directly influence therapeutic decision-making in clinical settings. In addition to individual markers, the field of biomarker discovery is progressing through various complementary categories. Imaging-based biomarkers, including FAP-targeted PET tracers, facilitate non-invasive measurement of stromal burden and therapeutic response [96]. Biomarkers that are soluble, like IL-6 and extracellular vesicles produced by CAFs, show a correlation with resistance phenotypes and might be useful for serial monitoring [63, 97]. Spatial and single-cell multi-omics methodologies are elucidating CAF niches with remarkable accuracy, associating subsets with immune evasion or angiogenesis [15, 20, 53]. Finally, functional biomarkers such as metabolic dependencies and pathway activity signatures might help in stratifying patients for reprogramming compared to depletion strategies [24].

The integration of these biomarker modalities into clinical trials is critical for enabling adaptive, subtype-specific enrolment and for establishing stromal endpoints alongside traditional tumor metrics. By incorporating biomarkers as potential stratification instruments and secondary endpoints in trials, we can achieve subtype-specific enrolment and gain a better understanding of stromal contributions to therapy responses, which is a crucial step towards adaptive trial designs that are guided by CAFS. This shift in paradigm alters the perception of CAFs, viewing them not as rigid adversaries but as flexible populations that can be therapeutically directed, thus advancing the domain of precision stromal oncology.

#### CAF plasticity and stromal reprogramming as the future paradigm

A key conceptual evolution is shifting the understanding of CAFs from rigid adversaries to flexible, reprogrammable populations. Accumulating evidence points to their dual capacities: while some subsets exhibit immunosuppressive and tumor-promoting characteristics, others could potentially restrict tumor growth or uphold tissue homeostasis [3, 59]. This duality calls for tactics that modify, rather than solely abolish the stroma.

Newly developed reprogramming techniques encompass nuclear receptor ligands, including VDR agonists and retinoids, which promote quiescence and restore matrix production [25, 26]. Epigenetic modulators can transition fibroblasts from a tumor-promoting state to a tumor-restraining state [39]. Metabolic reprogramming, including interventions aimed at CAF glycolysis or lactate management, provides additional avenues to transform the stromal environment [24, 45].

Risks remain substantial. Clinical observations in pancreatic cancer have indicated that extensive depletion of CAFs may lead to the emergence of more aggressive tumor phenotypes [18, 19]. Likewise, the modulation of off-tumor fibroblasts may jeopardize wound healing and tissue repair. Yet, the potential is equally substantial: by converting fibroblasts into immunostimulatory or tumor-restraining allies, it may be possible to turn the tumor’s architecture into a therapeutic partner. Additionally, these insights collectively suggest a future-oriented roadmap for “precision stromal oncology,” where the diversity of CAFs is harnessed to create rational, context-specific, and long-lasting therapeutic approaches.

In addition to phenotypic switching, a new aspect of CAF plasticity is their function as metabolic coordinators within the tumor-immune-stromal ecosystem. CAFs play a crucial role in regulating nutrient flows, such as lactate transport, amino acid exchange, and lipid metabolism, which subsequently influence immune suppression, angiogenesis, and responses to therapy [24, 45]. These metabolic activities are linked with classical CAF signaling mechanisms, illustrating that stromal modulation is not just about structure or immunity, but is also significantly bioenergetic. Integrating this metabolic angle into reprogramming strategies yields a more integrated perspective on CAF-directed interventions and bolsters the case for precision stromal oncology.

**Fig. 7:**
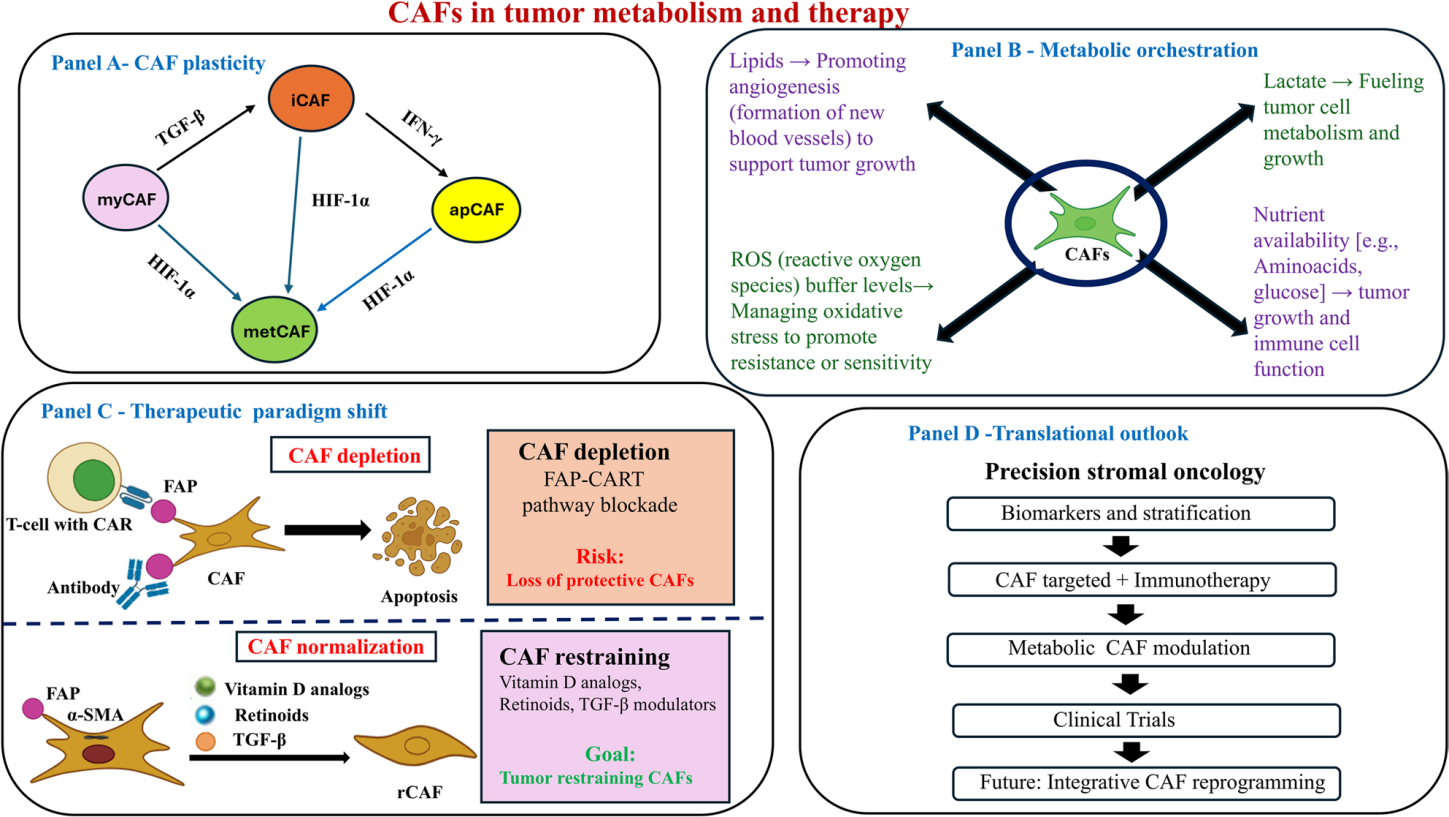
Conceptual framework for CAFs as metabolic orchestrators and therapeutic targets in the tumor-immune-stromal ecosystem. The figure illustrates the multifaceted roles of CAFs, including: (**A)** CAF plasticity: CAF subtypes (e.g., myofibroblastic, inflammatory, antigen-presenting) dynamically interconvert, and myCAFs, iCAFs, and apCAFs can convert to metabolic CAFs (metCAFs) in response to environmental cues and therapies [3, 5, 16, 20, 24, 37, 41–43, 45, 59–62]. (**B)** Metabolic Orchestration by CAFs in the TME. Schematic representation of CAFs regulating key processes in the tumor microenvironment through metabolic modulation. CAFs influence angiogenesis (lipids), tumor cell metabolism and growth (lactate), oxidative stress management (ROS buffer), and immune cell function (amino acids), promoting a favorable environment for tumor progression [24, 45, 60, 61, 79]. (C) Therapeutic paradigms: CAF-directed interventions span depletion strategies versus reprogramming approaches, highlighting their double-edged roles as tumor promoters and potential restrainers [5, 18, 19, 25, 26, 66, 98]. (D) Translational pipeline: Integration of CAF modulation with immuno-oncology, biomarkers, and patient stratification enables the vision of precision stromal oncology [5, 11, 101, 108, 124]

An integrative conceptual framework is needed to unify CAF biology with translational oncology. As represented in Fig. 7, CAFs operate as versatile and metabolically dynamic facilitators of the tumor-immune-stromal ecosystem.

The duality of their function as tumor-promoting or tumor-restraining cells is crucial to the evolution of new therapeutic models, which are shifting from non-specific depletion to precise reprogramming approaches that are integrated with immuno-oncology and biomarker-focused interventions.

The implementation of precision stromal oncology in clinical environments faces numerous translational challenges. Although the conceptual framework of precision stromal oncology is advanced, several practical and systemic barriers hinder its clinical use. The standardization of multi-omic CAF biomarkers among various institutions is inconsistent, due to differences in spatial transcriptomic platforms and data integration methods [125]. Adaptive clinical trial designs incorporating stromal endpoints require adaptable regulatory frameworks that can facilitate real-time biomarker stratification [55]. The technical variability inherent in multi-omic platforms introduces challenges to data normalization and the consistency of results across various institutions [22, 125]. Economic and logistical obstacles hinder the implementation of adaptive, biomarker-driven clinical trial designs, which require continuous data integration and specialized infrastructure [126]. Furthermore, the financial and infrastructural expenditures linked to the adoption of multi-omic profiling in standard oncology practice restrict access. Additionally, the absence of a consensus on validated surrogate endpoints for stromal normalization complicates the regulatory approval of stromal-targeting agents. Regulatory ambiguity remains in place concerning the safety assessment of stromal-targeting agents that impact both cancerous and physiologically important fibroblast populations [126, 127]. These systemic challenges underscore the importance of precision stromal oncology progressing alongside advancements in regulatory, bioinformatic, and clinical infrastructures. This entails the setup of large-scale data-sharing partnerships, standardized computational workflows, and harmonized regulatory systems to support extensive implementation [15, 27, 82].

### Future outlook: precision stromal oncology

the field is moving into a new era of precision stromal oncology, where the diversity of CAFs is harnessed rather than disregarded. Cutting-edge multi-dimensional technologies such as spatial multi-omics, AI-based CAF taxonomy, and organoid co-culture models are ready to create actionable stromal maps for tailored therapy development for patients. From a clinical perspective, this vision involves the selection of patients based on biomarkers, customizing interventions according to the specific CAF signatures associated with different tumor types [36, 63].

**Fig. 8:**
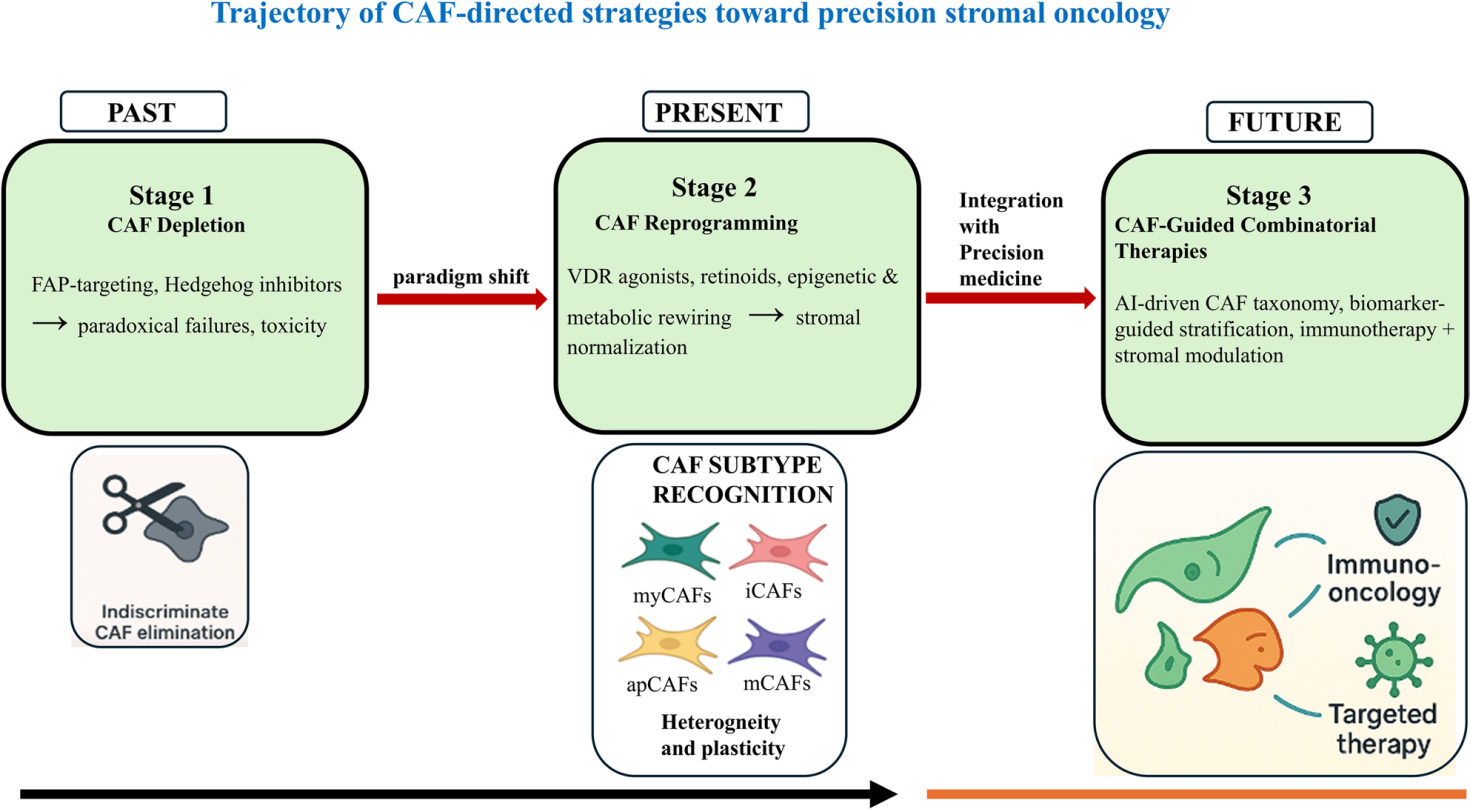
Roadmap of CAF-directed therapeutic strategies. A schematic representation of the evolution from indiscriminate CAF targeting to precision stromal modulation, showcasing advancements in targeted and effective approaches that highlight key strategies and emerging paradigms, depicting CAFs as reprogrammable, plastic allies rather than static enemies. Early efforts focused on indiscriminate CAF depletion (e.g., FAP-targeting, Hedgehog inhibition), which often produced paradoxical outcomes such as accelerated tumor progression and systemic toxicity [18, 19, 64, 95, 99]. Subsequent approaches emphasized CAF reprogramming using vitamin D receptor agonists, retinoids, and epigenetic or metabolic rewiring to normalize fibroblast function while preserving stromal architecture [24–26, 39, 66, 102]. The emerging frontier is CAF-guided combinatorial therapies, which leverage AI-driven CAF taxonomy, biomarker-guided patient stratification, and integration with immuno-oncology or targeted agents to achieve precision stromal modulation [29, 78, 100, 102, 105, 114, 115, 128]. This staged trajectory underpins the paradigm of precision stromal oncology

Conceptually, the trajectory of CAF-targeting is shifting from:

1. CAF eradication (direct depletion, resulting in mixed outcomes).
2. CAF reprogramming (normalization and subtype modulation).
3. CAF-guided combination therapies (integrating stromal modulation with immuno-oncology, targeted therapy, or virotherapy) as indicated in Fig. 8.

The developing knowledge of CAFs frames them as versatile partners that can be employed and reprogrammed to enhance cancer therapies through precision-guided approaches and combinatorial methods, converting them from challenges to therapeutic allies. This shift in paradigm highlights the increasing acknowledgment of stromal biology as fundamental to therapeutic responses, thereby facilitating the development of more effective, long-lasting, and contextually relevant treatments. Striking a careful balance between efficacy and safety is imperative, leading oncology towards a more integrated approach where stromal biology guides therapeutic strategies. The conceptual trajectory of CAF-targeting is evolving from: (i) the eradication of CAFs, which in certain contexts has paradoxically accelerated tumor progression [18], to (ii) strategies for reprogramming CAFs that aim to normalize or redirect fibroblast activity [3, 24], and (iii) combination therapies guided by CAFs that integrate stromal modulation with immuno-oncology or targeted therapies. Ultimately, the measure of success will not be confined to mechanistic insights but will also encompass observable improvements in patient survival and quality of life, attainable only when stromal endpoints and biomarker-guided stratification are included in the design of clinical trials [82].

## Discussion

The TME is not merely a passive environment; rather, it constitutes a dynamic ecosystem in which CAFs serve as crucial architects of malignant progression. Expanding upon the translational insights presented in (Section Translational outlook), our synthesis highlights CAFs as key regulators of tumor growth, immune evasion, and resistance to therapy. This section recontextualizes the unresolved inquiries in CAF biology and the therapeutic opportunities they present by incorporating ECM remodeling, paracrine signaling, phenotypic heterogeneity, and therapy response.

This discourse intricately combines evidence from (Sections CAFs in the Tumor Microenvironment to Translational outlook). Section CAFs in the Tumor Microenvironment has established the origins, phenotypes, and markers of CAF [5, 14, 15]; The mechanisms discussed in (Section Mechanisms of CAF-Mediated Tumor Progression) included ECM remodeling, cytokines, immunomodulation, angiogenesis, invasion, and resistance [12, 43, 68]; (Section Therapeutic Interventions Targeting CAFs) depicted strategies shifting from depletion to reprogramming [18, 25, 28]; and (Section Translational outlook) evaluated translational pipelines and biomarkers [23, 108, 129]. Here, we integrate these threads, address contradictions, and emphasize CAF plasticity as the central conceptual and translational axis [3, 20].

### ECM remodeling and paracrine coupling

#### Context

CAF-driven ECM remodeling results in collagen stiffening, an increase in density, and modifications to biochemical properties, which in turn facilitate invasion, limit drug penetration, and influence mechanical signaling [5, 12]. The findings from single-cell proteomic mapping reveal that alterations in the matrix are linked to resistance phenotypes and T cell exclusion in greater than 40% of pancreatic and breast tumors [24]. Recent advancements in spatial multi-omics have further identified a subset of F5-CAF that localizes near tumor nests in hepatocellular carcinoma, which is directly associated with cancer stemness and unfavourable prognosis [124].

**Contradiction**

Previously, ECM modifications were viewed as passive fibrosis. Yet CAFs secrete cytokines such as IL-6 and TGF-β, which trigger mechanotransduction pathways that enhance ECM rigidity, thereby obscuring the distinction between structural integrity and signaling [16, 43, 55].

#### Synthesis

 The process of ECM remodeling and paracrine signaling constitutes a self-reinforcing biophysical–biochemical circuit. The stiffening of the matrix initiates mechanotransduction and cytokine programs, which in turn enhance matrix remodeling, creating resistance niches [3, 12, 17]. This feedback mechanism elucidates the ineffectiveness of interventions targeting a single pathway and underscores the necessity for strategies that target dual pathways.

#### Open research question

Can combined inhibition of mechanotransduction and cytokine circuits effectively counteract CAF-driven resistance in vivo without disrupting stromal homeostasis [3, 17]?.

### CAF heterogeneity and functional duality

#### Context

The diversity of CAF was emphasized in (Section CAFs in the Tumor Microenvironment), covering myofibroblastic, inflammatory, antigen-presenting, and metabolic subtypes [14, 20]. Large-scale profiling of single cells from over 50,000 fibroblasts has substantiated that these subsets remain consistent across tumor types, indicating that inflammatory CAFs are abundant in immune-excluded tumors and that antigen-presenting CAFs are positioned adjacent to tertiary lymphoid structures [15, 23]. A pan-cancer atlas has further characterized senescence-associated cancer-associated fibroblasts (senesCAFs) and has proposed the SCRS signature as a predictive biomarker in the context of neuroblastoma [130]. Research indicates that in gastric cancer, apCAFs are linked to the response to immunotherapy, highlighting their significance as protective biomarkers [129].

#### Contradiction

 Despite this diversity, CAFs have historically been viewed as consistently tumor-promoting due to their roles in fibrosis, angiogenesis, and immune evasion. However, contradictory evidence suggests that the depletion of CAFs may accelerate tumor growth, implying that certain subsets may inhibit rather than encourage malignancy [18, 19].

#### Synthesis

CAFs serve as regulators that depend on their context. TGF-β promotes myofibroblastic programs that may have tumor-restraining effects, whereas IL-1/NF-κB stimulates inflammatory CAFs that contribute to tumor promotion [20, 55]. The significance of spatial positioning cannot be overstated: perivascular CAFs enhance the process of vascularization, whereas CAFs located at the borders contribute to immune exclusion [23]. This paradox is clarified when CAFs are perceived as versatile actors whose roles are shaped by the microenvironmental context. Effective translation requires aligned classifications through multimodal atlases and AI-facilitated taxonomies [23, 128].

#### Open question

 One of the main challenges is the lack of unified classification systems that can effectively differentiate tumor-promoting CAFs from tumor-restraining CAFs across different types of cancer. In what ways can multi-omic signatures be transformed into clinically applicable taxonomies, supported by biomarkers such as FAP-PET imaging [108] and proteomic classifiers [128]?.

### Mechanistic interplay: hierarchy or redundancy

#### Context

The activity of CAF is primarily influenced by two key pillars: ECM remodeling and cytokine-mediated signaling. The extent to which each of these factors contributes to tumor progression is still a topic of debate [5, 12, 43]. In PDAC, the rigidity of the extracellular matrix develops ahead of immune exclusion, whereas in breast cancer, cytokine-induced inflammation is the leading factor [12, 64]. Research utilizing spatial multi-omics in HCC has revealed that F5-CAFs co-localize with both stemness programs and immunosuppressive niches [124].

**Contradiction**

Some models indicate a hierarchical order in which ECM stiffening takes place before and shapes cytokine signaling, as evidenced in breast cancer [131] and pancreatic cancer, where mechanical stress initiates FAK–YAP signaling prior to the activation of inflammatory circuits [48]. Conversely, some contend that cytokine programs may function autonomously as key drivers, with IL-6, CXCLs, and TGF-β facilitating tumor advancement even in minimally fibrotic stroma [43, 68]. However, data derived from mechanotransduction investigations reveal redundancy and feedback loops, where the stiffening of ECM driven by YAP/TAZ and the secretion of cytokines support one another [49, 55].

#### Synthesis

Tumor-type-specific hierarchies are found within a framework that is redundant. Emerging research suggests that these mechanisms are interdependent rather than in competition with one another. The stiffening of the ECM facilitates mechanotransduction, which increases cytokine secretion, while cytokines further activate fibroblasts and promote additional ECM deposition [14, 15, 20]. It is important to emphasize that context is a key factor: Desmoplastic cancers are largely ECM-driven [18, 19], while inflammatory cancers are more dependent on cytokine signaling [43, 68]. Moreover, redundancy provides insight into the escape routes of resistance and explains why the inhibition of a single pathway can often lead to the activation of another, thereby causing therapeutic resistance. This stratified perspective offers a justification for tumor-targeted intervention strategies informed by perturbational experiments in organoid-CAF-immune models.

#### Open question

Is it possible for therapies that target both mechanotransduction and cytokine pathways at the same time to inhibit compensatory escape and achieve sustained efficacy across multiple cancer types [24, 53]?.

### Therapeutic contradictions: Depletion, Reprogramming, or combination

**Context**

Therapeutic approaches encompass depletion (anti-FAP), reprogramming (vitamin D analogs, retinoids), metabolic targeting, and CAF-guided combinations [25, 26, 28, 98].

**Contradiction**

Depletion leads to the removal of both protective and pathogenic CAFs, occasionally resulting in adverse outcomes; reprogramming can achieve stroma normalization, though it may be partial; combination strategies appear promising but add layers of complexity [18, 19, 28].

**Synthesis**

Precision modulation is more effective than blunt depletion. A recent example of this is the inhibition of NNMT, which successfully reprogrammed CAF metabolism, lowered immunosuppression, and reinstated checkpoint blockade. This highlights that focusing on plasticity through reprogramming, ideally within logical combinations, yields a safer and more lasting benefit than blunt depletion [66].

#### Open question

Are CAF-guided combinational therapies capable of achieving sustainable responses without compromising stromal homeostasis [28, 49]?.

### Explaining translational failures and future safeguards

**Context**

Despite significant preclinical potential, the clinical trials involving sibrotuzumab (anti-FAP) and Hedgehog inhibitors demonstrate a gap between preclinical models and patient responses [95, 99].

**Contradiction**

Why have strategies that worked well in animal models not translated successfully to patient outcomes? In murine systems, the depletion of stromal tissue led to improved drug delivery and a reduction in tumor burden [18, 19, 132]. Yet, in human cases, anti-FAP therapy did not yield any survival benefits [95], and the inhibition of Hedgehog paradoxically aggravated outcomes in PDAC [99]. This variation highlights the translational gap between regulated preclinical models and the heterogeneous tumors observed in patients.

**Synthesis**

The failures were attributed to (i) disregarding CAF heterogeneity, (ii) insufficient biomarkers for stratification, (iii) CAF plasticity that promotes adaptive escape, and (iv) pharmacokinetic and tumor-type specific stromal discrepancies. Moreover, it was shown that lactate produced by CAFs drives histone lactylation (H3K18la) and stabilizes NCAPG, thus promoting immune evasion and resistance to checkpoint therapies in gastric cancer [133]. These observations clarify why interventions that solely target immune checkpoints, without addressing CAF metabolism, are inadequate. Future precautions should involve biomarker-informed patient stratification, theranostic monitoring [108, 109], and humanized experimental systems to predict adaptive responses.

#### Open question

 Is it possible for adaptive, biomarker-stratified trials to address these shortcomings and realize translation [23, 109]?.

### Precision stromal oncology as a roadmap

#### Context

 In Sect. 5 translational outlook, it was highlighted that translational progress offers a basis for precision therapy. The existing CAF-focused translational tools now feature single-cell atlases, AI classifiers, organoid-CAF-immune models, and theranostic imaging [15, 20, 108, 128]. The emergence of predictive biomarkers such as SCRS signatures [130] and apCAF abundance [129] is noteworthy.

**Contradiction**

Historically, CAFs were considered static targets, with early depletion strategies (for instance, anti-FAP antibodies and Hedgehog pathway inhibitors) operating under the assumption that fibroblasts were entrenched in a tumor-promoting condition [18, 95, 102]. Nonetheless, a growing body of evidence indicates that CAFs exhibit significant dynamism and plasticity, shifting between myofibroblastic, inflammatory, and antigen-presenting states in response to therapeutic or microenvironmental signals [15, 20, 134]. This contradiction prompts an inquiry into how dynamic populations can be effectively incorporated into precision trial designs.

#### Synthesis

Precision stromal oncology delineates a strategic roadmap: the discovery of CAF states [15, 20], AI-driven classification [24, 128], validation within organoid-CAF-immune systems [53], stratification via FAP-PET [108, 109], and biomarker-based combination trials [27, 100]. Biomarkers like SCRS [130] and the levels of apCAF [129] highlight the role of stromal signatures in facilitating patient selection.

#### Open question

 Can this pipeline, from discovery to trial, effectively transform CAF biology into sustainable advantages for patients [27, 100, 130]?.

### Integration across Metabolism, Immunity, and Spatial niches

**Context**

CAFs govern metabolic flow, establish lactate-rich environments, inhibit immune cell presence, and influence vascular networks [24, 45]. Spatial multi-omics validates the presence of CAF neighbourhoods linked to both immune exclusion and supportive niches [58, 82].

**Contradiction**

Although CAFs are frequently portrayed as suppressors, they can also act as facilitators of immune infiltration in specific spatial contexts. Recent evidence suggests that they may stabilize vasculature and support protective immune responses. Recent research has shown a direct relationship between lactate produced by CAFs and immune evasion, underscoring the intersection of metabolic reprogramming with immune suppression [133].

**Synthesis**

As outlined in Sect. 6.1, ECM remodeling operates not only as a structural or signaling scaffold but also actively modifies nutrient diffusion, oxygen gradients, and metabolite availability, consequently forming bioenergetic niches that impact the functionality of tumor and immune cells [5, 12, 24]. The initial metabolic impacts of matrix remodeling align with the spatially structured functions of CAFs outlined here, emphasizing that the metabolic reprogramming facilitated by CAFs and their spatial arrangement are interdependent factors contributing to immune exclusion and resistance to therapy. Additionally, the way lactate-driven epigenetic remodeling fortifies NCAPG provides a mechanistic association between CAF metabolism and the shortcomings of immunotherapy [133]. The identification of these spatial-metabolic-immune signatures will enhance CAF-guided patient stratification for immunotherapy.

#### Open question

 Is it possible to develop integrated metabolic-immune-spatial CAF signatures as predictive classifiers for the outcomes of immunotherapy [82, 133]?.

### Plasticity as the central axis

#### Context

CAFs were initially viewed as fixed cell states; nevertheless, accumulating evidence demonstrates that they possess plasticity, enabling interconversion among myofibroblastic, inflammatory, antigen-presenting, and metabolic programs [15, 20, 23].

#### Contradiction

 CAF exhibits dynamic transitions based on microenvironmental and therapeutic stimuli [15, 20, 23].

#### Synthesis

 Plasticity addresses these contradictions. It elucidates the reasons behind the worsening of disease due to depletion, the feasibility of reprogramming, and the commonality of adaptive escape. Reviews underscore the dual functions in senescence, immune suppression, and resistance, further establishing plasticity as the primary determinant [59, 70, 134]. Therapeutic targeting should focus on stabilizing tumor-restraining CAFs or converting pathogenic states into quiescent states.

#### Open question

 Is it feasible to harness CAF plasticity in a predictable and stable manner in patients to achieve sustainable stromal control [25, 26]?.

By reinterpreting contradictions into synthesis and assimilating new insights from multi-omics, metabolism, and spatial mapping [40, 109, 124, 129], CAF biology is presented not as contradictory but as a framework for precision stromal oncology. The next obstacle, outlined in Future Directions and Challenges (Sect. 7), revolves around the question of whether CAF plasticity can be effectively and consistently employed in patients to ensure sustainable stromal control and extended therapeutic efficacy.

## Future directions and challenges

**Fig. 9:**
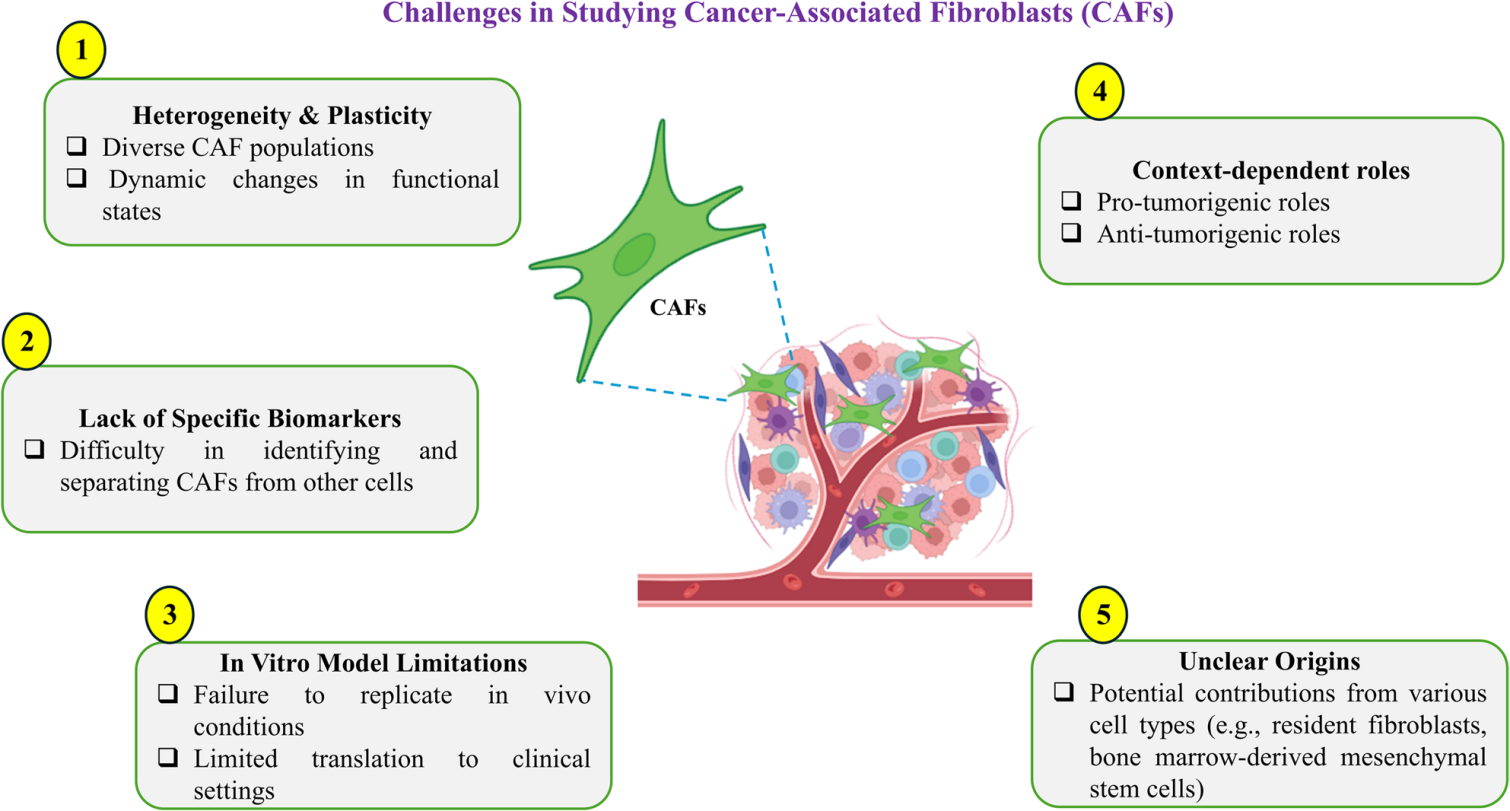
Challenges in Cancer-Associated Fibroblast (CAF) Research. Schematic representation of the complexities and hurdles in studying CAFs, including heterogeneity [3, 22, 40], lack of specific biomarkers [20, 29, 128], limitations of in vitro models [33, 36, 38], context-dependent roles [18, 19, 105], and unclear origins [5, 11, 81]. The critical unmet needs are threefold: (i) deriving lessons from failed strategies, (ii) creating classifiers that can distinguish between functional states of CAFs, and (iii) anticipating CAF plasticity in real-time to prevent adaptive escape

In the past decade, CAFs have been established as vital orchestrators of tumor progression and resistance to therapies. Despite the wealth of mechanistic insights available, the translation into sustainable therapies has been inadequate. Studying CAFs is challenging due to their complexity, heterogeneity, and dynamic interactions within the TME. Their context-dependent functions and lack of specific markers further complicate research and therapeutic targeting and major problems has highlighted in the Fig. 9.

### From illustrative maps to operational pipelines

 Multi-omics atlas has illustrated the diversity of CAFs across tumor types [15, 23], however, profiling by itself cannot guide therapy. The future calls for functional pipelines that go beyond simple description: candidate CAF states must be validated in organoid-CAF-immune co-cultures [53], transformed into biomarkers that can be identified in patients [108, 109], and woven into adaptive clinical trials. International consortia will be necessary to unify CAF taxonomies and produce reliable classifiers applicable to multiple cancers.

### Confronting translational failures

 The failure of Hedgehog inhibitors in pancreatic cancer [99] and sibrotuzumab in colorectal cancer [95] highlights persistent challenges: dependence on stromally deficient murine models, indiscriminate depletion of CAFs that eliminated protective subsets [18, 19], and the lack of predictive biomarkers. Recent trials, such as FAP-targeted PET imaging [108, 109] and early-phase CAF-CAR-T methodologies [28, 66, 135], reveal both feasibility and potential risks. These insights highlight the importance of ensuring that future trials are biomarker-driven, flexible in their design, and reinforced by longitudinal monitoring to accurately track stromal dynamics.

### Clinical urgency in refractory cancers

 CAF-rich tumors such as pancreatic ductal adenocarcinoma (PDAC), hepatocellular carcinoma (HCC), and triple-negative breast cancer (TNBC), still show resistance to checkpoint blockade and chemotherapy. In PDAC, desmoplastic CAFs form dense barriers that inhibit immune infiltration [12]. In HCC, the colocalization of F5-CAF subsets with stemness programs and immune exclusion suggests a poor prognosis [124]. In breast cancer, the cytokine networks driven by inflammatory CAFs support resistance to both endocrine therapy and immunotherapy [64]. These cases illustrate that addressing CAF biology is imperative, not merely optional, for overcoming therapeutic failures in solid tumors.

### Metabolic and immunometabolic vulnerabilities

 CAFs reshape metabolic landscapes, establishing lactate-abundant niches that suppress T-cell performance and induce histone lactylation, thus stabilizing oncogenic transcription [24, 133]. They also oversee lipid and arginine metabolism, which fosters T-cell exhaustion [45]. These functions establish CAF metabolism as a dual vulnerability: it both supports tumor cells and undermines immune responses. Future strategies should integrate spatial metabolomics and metabolic flux analysis into CAF classifications to pinpoint vulnerabilities specific to subsets, thereby creating opportunities for combined targeting with immunotherapy and metabolism-focused medications.

**Toward precision stromal oncology**

A crucial step will be the formulation of classifiers that can reliably distinguish tumor-promoting fibroblasts from tumor-restraining ones. Early candidates include the SCRS signature in neuroblastoma [130], the enrichment of antigen-presenting CAFs that predicts responses to immunotherapy in gastric cancer [129], and the activity of PDGF/HIF-1α that marks hypoxic niches [42]. Still, these biomarkers are not yet cohesive. AI-facilitated integration of multi-omic datasets [24, 128] and the implementation of deep learning in histopathology are surfacing as promising approaches for unified CAF classification. A progressive vision entails the creation of a “stromal passport,” which is a composite classifier that integrates FAP-PET imaging, multiplexed proteomics, and spatial immune signatures to categorize patients for stromal-targeted clinical trials [108, 109].

**Safeguarding translation through plasticity control**

The most significant obstacle is CAF plasticity, which allows fibroblasts to alter their phenotypes in response to therapeutic pressure [15, 20, 23]. In the absence of dynamic stratification, interventions may inadvertently remove protective CAFs or trigger compensatory states [18, 19]. Humanized co-cultures and patient-derived organoids provide platforms to capture fibroblast transitions that are specific to humans and absent in murine models [53, 82]. Moreover, real-time theranostics monitoring using FAP-PET imaging can effectively track stromal remodeling in patients [108, 109]. Incorporating these safeguards into the design of trials will be crucial for anticipating adaptive remodeling. It is only through addressing plasticity that stromal targeting can transition from experimental potential to lasting patient advantage.

### Limitations of current preclinical models

Despite notable progress, most current preclinical systems are inadequate in fully mimicking the cellular and spatial complexities of human CAF biology [3, 29]. Conventional two-dimensional co-culture assays deliver crucial insights into paracrine signaling; however, they do not effectively replicate the mechanical stiffness, ECM anisotropy, and gradient-dependent signals that typify the tumor stroma in vivo [136–138]. Similarly, organoids obtained from patients often lack fully matured immune and vascular compartments, thereby limiting their capacity to emulate the dynamic interactions among cancer-associated fibroblasts, immune cells, and endothelial cells that are essential for stromal activation and therapeutic response [31, 139, 140]. Despite the importance of murine models in mechanistic analysis, they differ from human CAF ontogeny and reveal species-specific distinctions in fibroblast lineage markers, cytokine networks, and immune architecture, which ultimately results in a constrained translatability of CAF-related strategies. This disparity emphasizes the need for caution when extrapolating results from murine studies to human scenarios. Therefore, further investigation is warranted to enhance the relevance of these findings [3, 29, 141]. These methodological constraints have resulted in a persistent disparity between promising preclinical outcomes and true clinical efficacy [3, 30]. The development of cutting-edge three-dimensional and humanized organoid-on-chip platforms that incorporate immune, stromal, and vascular components within matrices that are physiologically relevant provides a way to more effectively illustrate CAF heterogeneity, spatial arrangement, and temporal adaptability, consequently enhancing predictive capabilities for translational research [139, 142, 143].

## Conclusion

CAFs are now recognized as crucial architects of the TME, impacting progression, immunity, and therapeutic outcomes. The next ten years must focus on promoting precision stromal oncology to be on par with immuno-oncology as a vital component of cancer treatment. Progress will require three converging approaches: (i) establishing harmonized, systems-level taxonomies of CAF states through the integration of multi-omics and AI [15, 24]; (ii) exploiting metabolic and epigenetic dependencies to redirect fibroblasts instead of depleting them [23, 133]; and (iii) integrating stromal endpoints into adaptive, biomarker-stratified trials, supported by real-time monitoring using FAP-PET and spatial biomarkers [108, 109]. Above all, the plasticity of CAFs must be identified as the primary factor that determines both the failure and the potential for therapeutic success. Transforming CAFs from hindrances into therapeutic allies could be achieved by stabilizing protective states and neutralizing pro-tumor subsets. Just as how immuno-oncology transformed cancer treatment in the last decade, precision stromal oncology offers the blueprint for the next. One in which the future of cancer therapy will be determined not solely by malignant cells, but on our capacity to transform the stroma into a durable partner in cancer control.

## Supplementary Information

Supplementary Material 1. Table S1. [144–150]

## Data Availability

No datasets were generated or analysed during the current study.
