# Supplementary material for: Cancer-associated fibroblasts at the crossroads of tumor progression and therapy resistance: from heterogeneity to precision reprogramming
Source: J Egypt Natl Canc Inst. 2025 Dec 22;37:81. doi: 10.1186/s43046-025-00334-7 (PMC13313437; doi:10.1186/s43046-025-00334-7)
Supplement: Supplementary file 1 — Supplementary Material 1. Table S1. [144–150] [file 43046_2025_334_MOESM1_ESM.docx]

**Supplementary table 1.** CAF subtype markers across various cancers

CAF subtypes exhibit distinct marker profiles, which can vary depending on the cancer type. Identifying these markers is crucial for understanding CAF biology and developing targeted therapies. Understanding these markers can provide following insights into CAF function and cancer progression: 1. CAF heterogeneity and plasticity, 2. Role of CAFs in tumor progression and metastasis, 3. CAF-immune cell interactions, 4. Metabolic reprogramming in CAFs, 5. Therapeutic targeting of CAFs, 6. CAF biomarkers and diagnostics.

| **Subtype** | **Markers** | **Cancer type** | **References** |
| --- | --- | --- | --- |
| MyCAFs (Myofibroblastic CAFs) | α-SMA, FAP, PDGFRα/β, COL1A1 | **Lung cancer** | [144, 145] |
| iCAFs (Inflammatory CAFs) | IL-6, IL-8, IL-11, IL-17, CXCL12, PD-L1, High expression of inflammatory cytokines (e.g., IL-1β, TNF-α) |  |  |
| apCAFs (Antigen-presenting CAFs) | MHC class II molecules (e.g., HLA-DR, HLA-DP, HLA-DQ), CD40, CD74 |  |  |
| Metabolic CAFs | Glycolytic enzymes (e.g., HK2, PKM2), Metabolic regulators (e.g., HIF1α, c-Myc), Increased glucose uptake and lactate production, NNMT (Nicotinamide N-methyltransferase) |  |  |
| Senescent CAFs | Senescent CAFs, p16INK4a, p21CIP1, Senescence-associated secretory phenotype (SASP) factors (e.g., IL-6, IL-8) |  |  |
| Pro-angiogenic CAFs | VEGF, FGF, PDGF, PDPN, SEPP1, CLU, C3 and C7 |  |  |
| Vascular CAFs (vCAFs) | CD31 (PECAM1), VE-Cadherin (CDH5), VEGFR2 (KDR), Endothelial cell markers |  |  |
| MyCAFs (Myofibroblastic CAFs) | α-SMA, FAP, PDGFRα/β, COL1A1, ACTA2, POSTN, PDPN | **Breast cancer** | [144, 146] |
| iCAFs (Inflammatory CAFs) | IL-6, IL-8, CXCL12, PD-L1, HIF1A |  |  |
| apCAFs (Antigen-presenting CAFs) | HLA-DR, HLA-DP, HLA-DQ, CD40, CD74 |  |  |
| Metabolic CAFs | HK2, PKM2, HIF1α, c-Myc, NNMT |  |  |
| Senescent CAFs | SA-β-Gal, p16INK4a, p21CIP1, SASP factors (e.g., IL-6, IL-8) |  |  |
| Pro-angiogenic CAFs | VEGF, FGF, PDGF, PDPN |  |  |
| Vascular CAFs (vCAFs) | CD31 (PECAM1), VE-Cadherin (CDH5), VEGFR2 (KDR), Endothelial cell markers |  |  |
| MyCAFs (Myofibroblastic CAFs) | α-SMA, FAP, PDGFRα/β, COL1A1, POSTN | **Ovarian cancer** | [147, 148] |
| iCAFs (Inflammatory CAFs) | IL-6, IL-8, CXCL12, PD-L1 |  |  |
| apCAFs (Antigen-presenting CAFs) | HLA-DR, HLA-DP, HLA-DQ, CD40, CD74 |  |  |
| Metabolic CAFs | HK2, PKM2, HIF1α, c-Myc, NNMT |  |  |
| Senescent CAFs | SA-β-Gal, p16INK4a, p21CIP1, SASP factors (e.g., IL-6, IL-8) |  |  |
| Pro-angiogenic CAFs | VEGFA, FGF, PDGF, PDPN |  |  |
| Vascular CAFs (vCAFs) | CD31 (PECAM1), VE-Cadherin (CDH5), VEGFR2 (KDR), Endothelial cell markers |  |  |
| MyCAFs (Myofibroblastic CAFs) | α-SMA, FAP, PDGFRα/β, COL1A1, POSTN | **Colorectal cancer** | [144, 149] |
| iCAFs (Inflammatory CAFs) | IL-6, IL-8, CXCL12, PD-L1, COX-2 |  |  |
| apCAFs (Antigen-presenting CAFs) | HLA-DR, HLA-DP, HLA-DQ, CD40, CD74 |  |  |
| Metabolic CAFs | HK2, PKM2, HIF1α, c-Myc, NNMT |  |  |
| Senescent CAFs | SA-β-Gal, p16INK4a, p21CIP1, SASP factors (e.g., IL-6, IL-8) |  |  |
| Pro-angiogenic CAFs | VEGF, FGF, PDGF, PDPN |  |  |
| Vascular CAFs (vCAFs) | CD31 (PECAM1), VE-Cadherin (CDH5), VEGFR2 (KDR), Endothelial cell markers |  |  |
| MyCAFs (Myofibroblastic CAFs) | α-SMA, FAP, PDGFRα/β, COL1A1, POSTN | **Pancreatic cancer** | [144, 150] |
| iCAFs (Inflammatory CAFs) | IL-6, IL-11, FAP, CXCL1, CXCL12, |  |  |
| apCAFs (Antigen-presenting CAFs) | HLA-DR, HLA-DP, HLA-DQ, CD74, SLP1 |  |  |
| Metabolic CAFs | HK2, PKM2, HIF1α, high PLA2G2A |  |  |
| Senescent CAFs | SA-β-Gal, p16INK4a, p21CIP1, SASP factors (e.g., IL-6, IL-8) |  |  |
| Pro-angiogenic CAFs | VEGF, FGF, PDGF, PDPN |  |  |
| Vascular CAFs (vCAFs) | CD31 (PECAM1), VE-Cadherin (CDH5), VEGFR2 (KDR), Endothelial cell markers |  |  |

**Abbreviations:** α-SMA (Alpha-smooth muscle actin), ACTA2 (Actin alpha 2), CD31 (Cluster of differentiation 31 or PECAM1), CD40 (Cluster of differentiation 40), CD74 (Cluster of differentiation 74), CDH5 (Cadherin 5 or VE-Cadherin), CLU (Clusterin), COL1A1 (Collagen type I alpha 1), COX-2 (Cyclooxygenase 2), CXCL1 (C-X-C motif chemokine ligand 1), CXCL12 (C-X-C motif chemokine ligand 12), FAP (Fibroblast activation protein), FGF (Fibroblast growth factor), HIF1α (Hypoxia-inducible factor 1 alpha), HK2 (Hexokinase 2), HLA-DP (Human leukocyte antigen DP), HLA-DQ (Human leukocyte antigen DQ), HLA-DR (Human leukocyte antigen DR), IL-1β (Interleukin 1 beta), IL-6 (Interleukin 6), IL-8 (Interleukin 8), IL-11 (Interleukin 11), IL-17 (Interleukin 17), KDR (Kinase insert domain receptor or VEGFR2), MHC (Major histocompatibility complex), NNMT (Nicotinamide N-methyltransferase), PDGF (Platelet-derived growth factor), PDGFRα/β (Platelet-derived growth factor receptor alpha/beta), PD-L1 (Programmed death-ligand 1), PECAM1 (Platelet endothelial cell adhesion molecule 1 or CD31), PKM2 (Pyruvate kinase M2), PLA2G2A (Phospholipase A2 group IIA), POSTN (Periostin), p16INK4a (Cyclin-dependent kinase inhibitor 2A), p21CIP1 (Cyclin-dependent kinase inhibitor 1A), PDPN (Podoplanin), SA-β-Gal (Senescence-associated beta-galactosidase), SASP (Senescence-associated secretory phenotype), SEPP1 (Selenoprotein P), SLP1 (Secretory leukocyte peptidase inhibitor), TNF-α (Tumor necrosis factor alpha), VEGF (Vascular endothelial growth Factor), VEGFR2 (Vascular endothelial growth factor receptor 2 or KDR).
